# LRP1 influences trafficking of N-type calcium channels via interaction with the auxiliary α_2_δ-1 subunit

**DOI:** 10.1038/srep43802

**Published:** 2017-03-03

**Authors:** Ivan Kadurin, Simon W. Rothwell, Beatrice Lana, Manuela Nieto-Rostro, Annette C. Dolphin

**Affiliations:** 1 Department of Neuroscience, Physiology and Pharmacology, University College London, Gower Street, London, WC1E 6BT, UK

## Abstract

Voltage-gated Ca^2+^ (Ca_V_) channels consist of a pore-forming α1 subunit, which determines the main functional and pharmacological attributes of the channel. The Ca_V_1 and Ca_V_2 channels are associated with auxiliary β- and α_2_δ-subunits. The molecular mechanisms involved in α_2_δ subunit trafficking, and the effect of α_2_δ subunits on trafficking calcium channel complexes remain poorly understood. Here we show that α_2_δ-1 is a ligand for the Low Density Lipoprotein (LDL) Receptor-related Protein-1 (LRP1), a multifunctional receptor which mediates trafficking of cargoes. This interaction with LRP1 is direct, and is modulated by the LRP chaperone, Receptor-Associated Protein (RAP). LRP1 regulates α_2_δ binding to gabapentin, and influences calcium channel trafficking and function. Whereas LRP1 alone reduces α_2_δ-1 trafficking to the cell-surface, the LRP1/RAP combination enhances mature glycosylation, proteolytic processing and cell-surface expression of α_2_δ-1, and also increase plasma-membrane expression and function of Ca_V_2.2 when co-expressed with α_2_δ-1. Furthermore RAP alone produced a small increase in cell-surface expression of Ca_V_2.2, α_2_δ-1 and the associated calcium currents. It is likely to be interacting with an endogenous member of the LDL receptor family to have these effects. Our findings now provide a key insight and new tools to investigate the trafficking of calcium channel α_2_δ subunits.

Voltage-gated Ca^2+^ (Ca_V_) channels are multi-subunit complexes, containing a pore-forming α1 subunit, which determines the main functional and pharmacological attributes of the channel^1^. The high voltage-activated Ca_V_1 and Ca_V_2 α1 subunits are associated with two auxiliary subunits, both of which affect the trafficking and properties of these channels. The intracellular β subunit binds to the channels in the endoplasmic reticulum (ER), and protects the channel from ER-associated degradation^2^,^3^. In contrast, the mechanism by which the α_2_δ subunit enhances the functional expression of the channel complex is less well understood^4^,^5^.

Genes encoding four α_2_δ subunits have been identified (for review see ref. 6). We have shown that α_2_δ subunits are glycosylphosphatidylinositol (GPI)-anchored proteins^7^, which accumulate in detergent-resistant microdomains (DRMs), also termed lipid rafts^7^,^8^. This is confirmed for α_2_δ-1 in the recent high resolution structure of Ca_V_1.1^9^. We have also shown the Von Willebrand Factor A (VWA) domain of α_2_δ subunits to be crucial for the trafficking of the associated α1 subunits to the plasma membrane^4^, and for their ability to increase calcium currents and transmitter release^4^,^10^.

Both α_2_δ-1 and α_2_δ-2 bind to the anti-epileptic and anti-hyperalgesic drugs gabapentin and pregabalin^8^,^11^. Indeed, binding to α_2_δ-1 is essential for the therapeutic effect of these drugs in the alleviation of neuropathic pain^12^. We have shown that chronic application of gabapentinoids suppresses calcium currents by inhibiting α_2_δ-1 and α_2_δ-2 trafficking, and have proposed this as the mechanism of action of these drugs^13^,^14^,^15^.

Identification of the molecular mechanisms that control the trafficking of α_2_δ subunits is central to understanding their diverse roles in the regulation of Ca_v_α_1_ subunit plasma membrane localization^5^, in synaptic transmission^10^,^16^,^17^, as mediators of the therapeutic effects of the gabapentinoid drugs^12^, and in relation to their novel roles in synaptic function^5^,^18^,^19^.

Using bungarotoxin binding site (BBS)-tagged α_2_δ-2, we have previously observed constitutive endocytosis and recycling of the α_2_δ-2 subunit from recycling endosomes, in a rab11-dependent manner^13^. Since α_2_δ subunits are GPI-anchored^7^,^9^, these results invoke the existence of an adaptor protein to bridge the interaction between α_2_δ subunits and intracellular trafficking pathways.

Low density lipoprotein (LDL) receptor-related protein-1 (LRP1) is a member of a large LDL receptor family^20^, which also includes LDL receptor, very low density lipoprotein receptor, apoE receptor 2, glycoprotein 330 (gp330/megalin/LRP2) and LRP1B^21^. In addition, this protein family comprises LRP5, and LRP6, which are involved in Wnt signalling^22^. LRP1 is multifunctional receptor, interacting with number of protein ligands, most of which bind to the four clusters of cysteine-rich and EGF- repeats, termed ligand binding domains (LBDs). It mediates both forward trafficking and endocytosis of protein cargoes^21^. Binding of LRP1 ligands at the plasma membrane can also promote its assembly into co-receptor systems and initiate intracellular cell signaling cascades^23^.

We surmised that LRP1 would be a good candidate for a role in trafficking the α_2_δ subunits, since it is involved in forward trafficking of a number of other proteins, including β1-integrin^24^ and GPI-anchored PrP^25^, and in clathrin-dependent endocytosis^21^,^26^. It is also involved in the trafficking of glucose transporter-4, which is rapidly inserted into the plasma membrane from sub-membrane storage vesicles in response to insulin^27^. In the nervous system, LRP1 is present in synapses^28^, as is α_2_δ-1^15^, and neuronal deletion of the LRP1 gene results in a phenotype including neurodegeneration and synapse loss, and development of behavioral and motor abnormalities^29^,^30^. The interaction of LRP1 with specific ligands has also been implicated in neurite outgrowth^31^. LRP1 has also been shown to bind to thrombospondins with high affinity^32^, which is implicated in synapse formation^19^, and is able to bind more than one ligand at different sites^33^.

LRP1 and related proteins interact with Receptor-Associated Protein (RAP), a 39 kDa specific chaperone protein resident in the ER, which prevents interaction of LRP1 with its ligands until they reach the Golgi^34^. The relatively acidic lumenal environment of the Golgi causes protonation of key histidine residues, and a structural rearrangement ‘histidine switch’ of RAP, which leads to its dissociation from LRP1^35^. RAP has been shown to be about 2-fold down-regulated in dorsal root ganglion (DRG) neurons following nerve injury^36^, suggesting that the interaction of LRP1 (and other family members) with their ligands might be dynamically regulated by the availability of RAP.

In the present study we show that α_2_δ-1 is a ligand for LRP1, and their interaction is modulated by RAP. This interaction regulates the trafficking, post-translational processing, and function of α_2_δ-1 as a calcium channel subunit. Our findings now provide a key insight and new tools to investigate the trafficking and function of the calcium channel α_2_δ subunits.

## Results

### Interaction between LRP1 and α_2_δ subunits

We first examined whether LRP1 is able to interact with α_2_δ-1 subunits. LRP1 is a 600 kDa pro-protein that is cleaved by the Golgi-associated protease furin into an ~85 kDa light-chain and a ~515 kDa heavy-chain; the two polypeptides remaining non-covalently-associated. The heavy-chain contains four homologous LBDs, which were identified to mediate binding to most of the LRP1 interacting proteins^37^,^38^. Each domain can be expressed independently, fused with the common 85 kDa transmembrane domain^39^. These are termed mini-receptors (LRP1-m1–m4), and have been shown to mimic the effect of full-length LRP1^38^,^39^. Most of the data obtained here involves experiments with a construct consisting of the fourth LBD of LRP1 (LRP1-m4).

**Figure 1:**
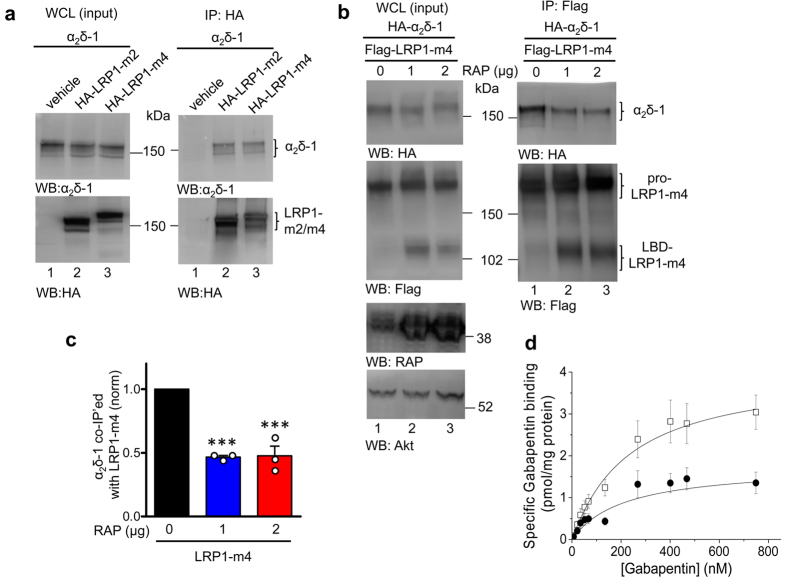
LRP1-m4 interaction with α_2_δ-1 is decreased by RAP. **(a)** tsA-201 cells were transfected with α_2_δ-1 (lanes 1–3), alone or with HA-LRP1-m2 (lane 2) or HA-LRP1-m4 (lane 3). Western blots showing: left panels, WCL; right panels, immunoprecipitate (IP) with HA Ab. Top panels: Western blot against α_2_δ-1 (α_2_δ-1 mAb), bottom panels: western blot against HA for HA-LRP1-m2 or -m4. The IP western blot has been stripped from α_2_δ-1 mAb and reblotted against HA. Samples were not deglycosylated. **(b)** tsA-201 cells transfected with HA-α_2_δ-1 and Flag-LRP1-m4 (lanes 1–3), without RAP (lane 1) or co-transfected with 1 μg or 2 μg RAP (lanes 2 and 3). Ca_V_2.2 and β1b were co-transfected in all conditions. Samples were not deglycosylated. Left panels: Input WCL; right panels, co-IP with anti-Flag Ab. Top panels: HA-α_2_δ-1 (HA Ab), middle panels: Flag-LRP1-m4 (Flag Ab), lower panels: RAP reblot (anti-LRP1/RAP Ab), and anti-Akt loading control. **(c)** Quantification of co-IP measured as a ratio of the HA-α_2_δ-1 bands in the co-IP, relative to the input WCL (including experiment shown in **(b)**), in the presence of Flag-LRP1-m4, and in the absence (black bar) or presence of the two concentrations of RAP shown in (**b**) (blue and red bars). Mean ± SEM and individual data for n = 3 experiments, each normalised to the control in the absence of RAP. *** *P* < 0.001, 1-way ANOVA and Bonferroni’s post-hoc test. **(d)** [^3^H]-gabapentin binding curves, using membrane fractions from transfected tsA-201 cells, for binding to α_2_δ-1 (open squares, n = 6) and α_2_δ-1 + LRP1-m4 + RAP (black circles, n = 6). Mean ± SEM data were fit by the Hill equation: K_D_ = 229.5 and 213.7 nM, respectively. B_max_ = 4.02 and 1.76 pmol/mg protein, respectively. Full blots for all figure parts are shown in Supplementary information.

The LRP1 mini-receptors were transiently expressed together with α_2_δ-1 in tsA-201 cells. In preliminary studies, we found LRP1 mini-receptors containing LBDs 2, 3 and 4 (HA-tagged LRP1-m2, -m3 and -m4) all co-immunoprecipitate with α_2_δ-1, whereas HA-LRP1-m1 showed less co-immunoprecipitation, as has also been found for other LRP1 ligands^39^. Figure 1a shows α_2_δ-1 interaction with HA-LRP1-m2 and HA-LRP1-m4 (data with HA-LRP1-m1 and -m3 not shown). We also co-expressed Flag-tagged LRP1-m4 together with HA-tagged α_2_δ-1 (HA-α_2_δ-1), and we used either anti-HA or anti-Flag antibodies on beads for pulldown. In both cases reciprocal co-immunoprecipitation was observed. Figure 1b shows immunoprecipitation of Flag-LRP1-m4 and co-immunoprecipitation of HA-α_2_δ-1. As expected, the reciprocal immunoprecipitation of HA-α_2_δ-1 also co-immunoprecipitated Flag-LRP1-m4 (data not shown). As a positive control for the co-immunoprecipitation we used Prion protein (PrP), which has previously been shown to interact with LRP1^25^ (data not shown). We have previously used the same constructs to demonstrate co-immunoprecipitation with another known LRP1 ligand, thrombospondin-4^40^.

### Interaction between α_2_δ-1 and LRP1-m4 is reduced by RAP

The ER-resident chaperone RAP binds at multiple sites on LRP1, and has a dual role. It antagonises premature binding of LRP1 with its ligands in the ER and also promotes LRP1 folding and trafficking to the Golgi where ligand interaction occurs^41^. Premature binding to LRP1 in the ER was reported for other known LRP1 ligands and is considered to be a general property of LRP1^42^. The interaction of α_2_δ-1 with LRP1-m4 was significantly reduced, but not prevented, by co-expression of RAP (Fig. 1b, compare lane 1 with lanes 2 and 3 in the right upper panels, quantified in Fig. 1c). The amount of RAP used (1 μg cDNA) was likely to be saturating, as doubling the amount of cDNA had no additional effect (Fig. 1b, compare lanes 2, and 3, quantification in Fig. 1c). Similar results were obtained with LRP1-m2 (data not shown), demonstrating that α_2_δ-1 binds indiscriminately to LBDs 2 and 4. As a negative control, instead of RAP we transfected the cells with GFP (Fig. 1b; lane 1; Fig. 1c), which demonstrated that the effect of RAP on the interaction is specific and it is not due to reduced protein expression. Thus the continuing interaction of LRP1-m2 and -m4 with α_2_δ-1 when RAP is co-expressed is likely to represent an interaction that is relevant to α_2_δ-1 trafficking.

### Interaction with LRP1 reduces the binding of gabapentin to α_2_δ subunits

We then examined the effect of LRP1-m4 co-expression on [^3^H]-gabapentin binding to α_2_δ-1. LRP1-m4 and RAP were co-expressed with α_2_δ-1 in tsA-201 cells. Saturation binding experiments analysing the binding of [^3^H]-gabapentin to membranes from cells expressing α_2_δ-1 and α_2_δ-1/LRP1-m4 demonstrate that LRP1-m4 does not alter the gabapentin binding affinity to α_2_δ-1 (Fig. 1d). The K_D_ for α_2_δ-1 alone was 180.2 ± 23.0 nM, and for α_2_δ-1/LRP1-m4 it was 230.0 ± 53.0 nM (n = 6, P > 0.05). In contrast, the presence of LRP1-m4 resulted in a 38% decrease in the measured B_max_ for [^3^H]-gabapentin binding to α_2_δ-1 from 3.63 ± 0.56 pmol/mg protein, to 2.06 ± 0.52 pmol/mg protein when LRP1-m4 was co-expressed with α_2_δ-1 (n = 6, *P* = 0.0129). This suggests that when α_2_δ-1 interacts with LRP1-m4, the association may occlude the gabapentin binding site.

### α_2_δ-1 and LRP1-m4 bind *in vitro*

**Figure 2:**
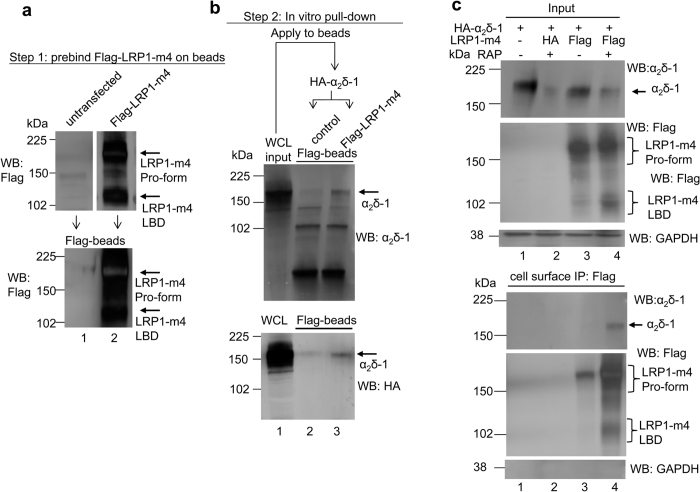
LRP1-m4 and α_2_δ-1 interact *in vitro.* **(a)** Western blot analysis of Flag-LRP1-m4 interaction with HA-α_2_δ-1. Samples were not deglycosylated. The top panels show aliquots from WCL prepared from untransfected- or Flag-LRP1-m4 and RAP-transfected tsA-201 cells (upper panel: Flag Ab), which were then applied to anti-Flag-agarose beads overnight to precipitate Flag-LRP1-m4. An aliquot of the beads with precipitated material was eluted to show bound Flag-LRP1-m4 (lower panel: Flag Ab). The remaining beads with pre-bound Flag-LRP1-m4 were used for the *in vitro* binding assay. **(b)** Lane 1 in top (anti-α_2_δ-1) and bottom (anti–HA) western blot panels corresponds to aliquots of the WCL from HA-α_2_δ-1 transfected tsA-201 cells, which were applied to beads with pre-bound Flag-LRP1-m4 (lane 3 in (**b**), equivalent to lane 2 in (**a**)), or untransfected cells (lane 2 in (**b**), equivalent to lane 1 in (**a**)). Arrows on right indicate α_2_δ-1 binding. **(c)** Cell surface co-IP from tsA-201 cells expressing HA-α_2_δ-1 without (lane 1) or with HA-LRP1-m4 (lane 2) or Flag-LRP1-m4 (lanes 3, 4), with or without RAP, as indicated, analysed by western blot. Samples were not deglycosylated. WCL input (top); cell surface co-IP with Flag Ab (bottom). For both WCL and IP: top panel: HA-α_2_δ-1 (α_2_δ-1 Ab); middle panel: Flag-LRP1-m4 (Flag Ab); bottom panel: samples ran on separate gel and reblotted against GAPDH loading control and control for cell surface IP. Representative of n = 3 experiments. Full blots for all figure parts are shown in Supplementary information.

Further evidence for the interaction between α_2_δ-1 and LRP1-m4 was obtained from *in vitro* interaction experiments. Lysates from tsA-201 cells expressing HA-α_2_δ-1 were applied either to beads loaded with pre-bound Flag-LRP1-m4, or to control beads pre-incubated with WCL from untransfected cells under identical conditions (Fig. 2a). After washing, HA-α_2_δ-1 was found attached to LRP1-m4 containing beads, but not to control beads (Fig. 2b, lane 3).

### LRP1-m4 and α_2_δ-1 are associated on the cell surface in the presence of RAP

Since an interaction between α_2_δ-1 and LRP1-m4 is maintained in the presence of RAP (Fig. 1b,c), we hypothesised that the two proteins may remain associated on the cell surface. To test this, we applied Flag antibody to intact tsA-201 cells transfected with α_2_δ-1 and either Flag-LRP1-m4 or HA-LRP1-m4, with or without RAP; then incubated cells at 4 °C to prevent endocytosis, and immunoprecipitated Flag-LRP1-m4, following cell lysis. We found more immunoprecipitation of LRP1-m4 from the cell surface in the presence of co-transfected RAP, than in its absence (Fig. 2c, lower panel, lane 4 compared to lane 3). We also found that α_2_δ-1 co-immunoprecipitated with cell surface Flag-LRP1-m4, only in cells co-transfected with LRP1-m4 and RAP, and was not detectable without RAP (Fig. 2c, lower panel, lane 4 compared to lane 3). This also agrees with one of the main roles of RAP being to promote folding and trafficking of LRP1 and its ligands to the cell surface.

### LRP1 binds anchorless α_2_δ-1ΔC *in vitro*

**Figure 3:**
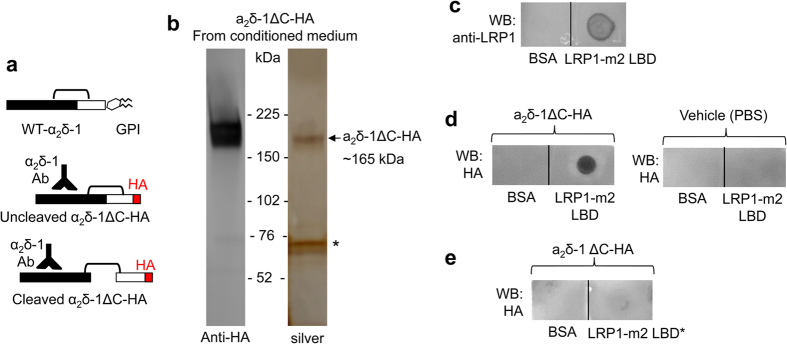
Purified α_2_δ-1ΔC binds to purified LRP1-m2, but not to BSA immobilised by dot blot on nitrocellulose membranes. **(a)** Cartoon showing α_2_δ-1ΔC construct with a C-terminal HA tag (middle and bottom) in place of the GPI anchor signal sequence present in WT α_2_δ-1 (top). **(b)** α_2_δ-1ΔC-HA affinity-purified on an HA-agarose-column from conditioned medium of transfected tsA-201 cells, visualised by western blot with HA Ab (left). Right: silver stain of the gel with the same material showing α_2_δ-1ΔC-HA (MW ~165 kDa) and an unspecific band (*; ~70 kDa). **(c)** LRP1-m2 LBD (0.2 μg, left) or BSA (0.2 μg, right) were immobilised on a nitrocellulose membrane and the protein was visualised by anti-LRP1 Ab. **(d)** LRP1-m2 LBD (0.2 μg) or BSA (0.2 μg) were immobilised on nitrocellulose membranes and incubated with either 50 nM purified α_2_δ-1ΔC-HA (left panel), or vehicle buffer (right panel), followed by visualisation of the bound α_2_δ-1ΔC-HA with anti-HA Ab, as described in Methods. Representative of n = 3. **(e)** The structure of LRP1 LBDs is crucial for ligand binding, as pre-treatment of purified LRP1-m2 LBD in buffer with 2% SDS and 100 mM DTT, to denature the protein and disrupt disulphide bonds (LRP1-m2 LBD*), resulted in less α_2_δ-1ΔC-HA binding. Full blots for all figure parts are shown in Supplementary information.

We then utilised a soluble anchorless α_2_δ-1 construct (α_2_δ-1ΔC, Fig. 3a), which is truncated prior to the C-terminus so that it is not GPI-anchored, and has previously been characterized to be in part secreted as a soluble protein^43^. We found, using a dot blot technique previously employed to demonstrate interactions with LRP1^44^, that soluble α_2_δ-1ΔC-HA protein purified from conditioned medium (Fig. 3b; arrow) and purified soluble LRP1-m2 LBD protein immobilised on nitrocellulose membrane (Fig. 3c), interact *in vitro*, as shown by the staining for α_2_δ-1ΔC-HA (Fig. 3d; left panel). An identical amount of bovine serum albumin (BSA) protein immobilised on the same membrane adjacent to LRP1-m2 did not bind to α_2_δ-1ΔC-HA, demonstrating the specificity of the interaction (Fig. 3d; left panel). As an additional control, membranes with immobilised BSA and LRP1-m2 LBD were treated under identical conditions except α_2_δ-1ΔC-HA was omitted, and the HA antibody signal was lost, showing that it is due to specific labelling of α_2_δ-1ΔC-HA by this antibody (Fig. 3d; right panel). Moreover, the interaction was dependent on structural features of LRP1-m2 LBD, because denaturation of the protein prior to its immobilisation on the membrane resulted in a decrease of the interaction with α_2_δ-1ΔC-HA (compare Fig. 3e with the left panel of Fig. 3d).

### Interaction between endogenous LRP1 and α_2_δ-1 in DRMs

**Figure 4:**
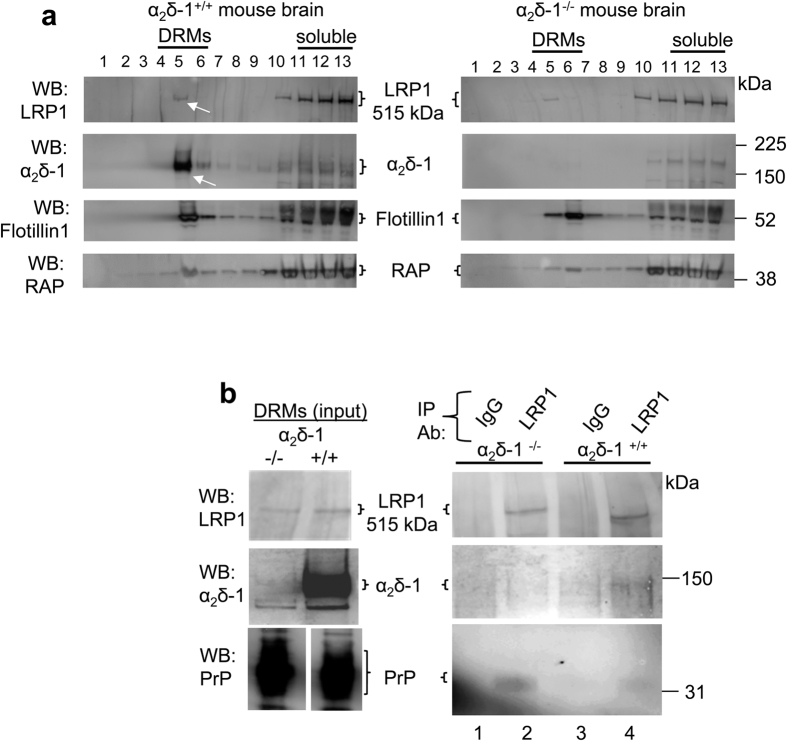
Localization and co-immunoprecipitation of α_2_δ-1 and LRP1 in DRMs. **(a)** Sucrose gradient fractions prepared from α_2_δ-1^+/+^ (left panel) and α_2_δ-1^−/−^ mouse brain (right panel), showing that LRP1 was mainly concentrated in the detergent-soluble fractions (top panel, lanes 11–13), although a small proportion of LRP1 is present in DRM fraction 5 (white arrow); whereas α_2_δ-1 is concentrated in DRM fraction 5 (second panel; left, white arrow), and is absent from α_2_δ-1^−/−^ brain (second panel; right). The third panel shows the DRM marker flotillin-1 (lanes 4–6), and the bottom panel shows RAP, which was also identified by the anti LRP1/RAP polyclonal Ab. 5 μl aliquots of the sucrose gradient fractions are loaded in each lane. **(b)** Left panel: input for IP are peak DRM fractions from α_2_δ-1^−/−^ (left lane) and α_2_δ-1^+/+^ (right lane) mouse brains. Right panel: IP with unrelated rabbit IgG (lanes 1 and 3) or LRP1 rabbit polyclonal Ab (lanes 2 and 4) from α_2_δ-1^−/−^ (lanes 1 and 2) and α_2_δ-1^+/+^ (lanes 3 and 4) DRMs. Western blots: top panel: LRP1; middle panel: α_2_δ-1, showing co-immunoprecipitation of α_2_δ-1 (lane 4); bottom panel: PrP (positive control for co-IP, lanes 2 and 4). Note that the contrast of the western blot shown in the right panel has been enhanced to detect low signals. Representative of n = 2 independent experiments. Full blots for all figure parts are shown in Supplementary information.

Both α_2_δ proteins^7^,^8^ and PrP^45^ are GPI-anchored DRM-associated proteins, and both interact with LRP1. We therefore performed experiments using material from mouse brain, and found some LRP1 in DRMs from WT mouse brain, although most was in the soluble fractions (Fig. 4a, left top panel, white arrow). The same was true for RAP (Fig. 4a, bottom panels). Therefore, since most endogenous α_2_δ-1 is present in DRMs (Fig. 4a, left second panel, white arrow), we decided to use mouse brain DRMs as the input, to attempt to co-immunoprecipitate native α_2_δ-1 with LRP1. As controls, brains from α_2_δ-1^−/−^ mice were included (Fig. 4a, right panels), as well as control immunoprecipitation with an irrelevant IgG, and also co-immunoprecipitation of the known LRP1 ligand PrP. LRP1 was immunoprecipitated with LRP1 Ab (Fig. 4b; top right panel, lanes 2 and 4).

The small amount of LRP1 in DRMs co-immunoprecipitated with a correspondingly small fraction of the total α_2_δ-1 in DRMs from α_2_δ-1^+/+^ mice (Fig. 4b; middle panel, lane 4). As a control, α_2_δ-1 was not co-immunoprecipitated with an irrelevant IgG (Fig. 4b; middle panel, lane 3), or in identical experiments using α_2_δ-1^−/−^ brain DRMs (Fig. 4b; middle panel, lanes 1 and 2). A similarly small fraction of PrP, relative to the total PrP that localised to DRMs, was co-immunoprecipited with LRP1 in parallel (Fig. 4b, bottom panel, lanes 2 and 4), which is again in line with the minor localisation of LRP1 in DRMs.

### RAP increases the cell surface expression of α_2_δ-1 in the presence of LRP1-m4

**Figure 5:**
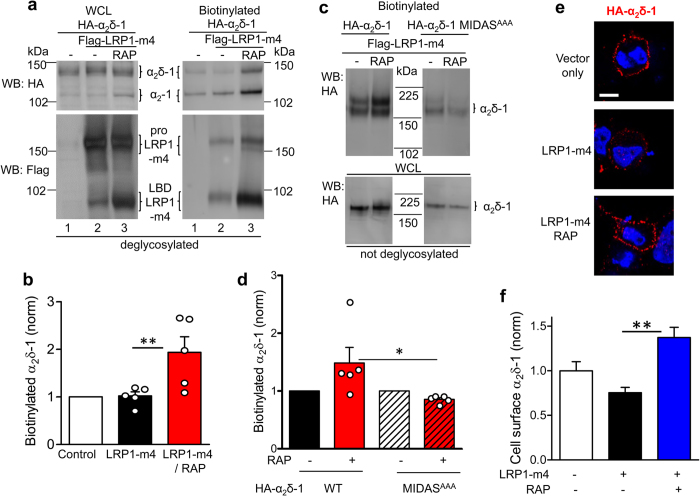
RAP promotes the cell surface expression of both LRP1-m4 and α_2_δ-1, which remain associated at the cell surface. **(a)** tsA-201 cells expressing HA-α_2_δ-1, without or with Flag-LRP1-m4 and RAP, were cell surface biotinylated and analysed by western blot. Samples were deglycosylated with PNGase-F. Left panel: WCL; right panel: cell-surface-biotinylation. Top panel: HA-α_2_δ-1 (HA Ab), bottom panel: Flag-LRP1-m4 (Flag Ab). Both pro-LRP1-m4 and cleaved LBD-LRP1-m4 (indicated) are present on the cell surface. **(b)** Quantification of HA-α_2_δ-1 on cell surface expressed as a ratio of biotinylated fraction to input WCL for 5 experiments, including that shown in (**a**). The data are normalised to the control in each experiment, in the absence of LRP1-m4 or RAP (open bar), LRP1-m4 alone (black bar) and LRP1-m4 plus RAP (red bar). Data are mean ± SEM with individual data points, **P < 0.01 ANOVA and Bonferroni post-hoc test. **(c)** tsA-201 cells expressing Flag-LRP1-m4 with HA-α_2_δ-1 (left panel) or HA-α_2_δ-1 MIDAS^AAA^ (right panel), without (left lanes) or with RAP (right lanes). Samples were not deglycosylated. Top panel: cell surface-biotinylated α_2_δ-1; bottom panel WCL. **(d)** Quantification of α_2_δ-1 on cell surface for 5 experiments including that shown in (**c**), relative to control without RAP in each experiment, for LRP1-m4 and WT α_2_δ-1 without (black bar), or with RAP (red bar), or α_2_δ-1 MIDAS^AAA^, without (black and white hatched bar) or with, RAP (red and black hatched bar). Data are mean ± SEM with individual data points, *P < 0.05 ANOVA and Bonferroni post-hoc test. **(e)** tsA-201 cells were transfected with HA-α_2_δ-1 alone (top panel), plus LRP1-m4 (middle panel) or LRP1-m4 and RAP (bottom panel) and cell surface-labelled with rat HA Ab (red). The images are merged with DAPI (blue). Scale bar 10 μm. **(f)** Bar chart (mean ± SEM) showing effect on HA-α_2_δ-1 cell surface expression of LRP1-m4 (black bar, n = 184 cells); and LRP1-m4 plus RAP (blue bar, n = 149 cells), normalised to control HA-α_2_δ-1 alone (open bar, n = 120 cells), from n = 2 independent transfections. **P < 0.01, 1-way ANOVA and Bonferroni’s post-hoc test. For consistency with the functional experiments shown in Fig. 8, Ca_v_2.2 and β1b subunits were co-transfected in all the experimental conditions (**a–f**). Full blots in Supplementary information.

In agreement with the cell surface co-immunoprecipitation results shown in Fig. 2c, cell surface biotinylation experiments provided evidence that RAP markedly increased the amount of α_2_δ-1 on the cell surface in the presence of LRP1-m4 (Fig. 5a, upper right panel, lane 3 compared to lane 2; quantified in Fig. 5b). As shown previously^39^, the processing and surface expression of LRP1-m4 were increased in the presence of RAP, demonstrating its effects on the trafficking of LRP1 itself (Fig. 5a, compare lanes 2 and 3 in the bottom right panel). We also examined the cell-surface expression of a non-functional α_2_δ-1 construct with mutations in the metal ion-dependent adhesion site (MIDAS) motif of the VWA domain (α_2_δ-1-MIDAS^AAA^)^4^,^10^. We have previously shown that this mutant demonstrates reduced cell surface expression, compared to WT α_2_δ-1^46^. We found here that plasma membrane expression of this mutant is not increased by co-expression of LRP1-m4 and RAP, unlike WT α_2_δ-1 (Fig. 5c, right panel; quantified in Fig. 5d). This suggests that during its trafficking α_2_δ-1 may require an intact VWA domain to be affected by LRP1.

Furthermore, in agreement with the cell surface biotinylation results, when tsA-201 cells were transfected with HA-α_2_δ-1 and LRP1-m4, with or without RAP, cell surface expression of HA-α_2_δ-1, detected immunocytochemically, was also increased by the presence of RAP (Fig. 5e, quantified in Fig. 5f).

### RAP increases secretion of anchorless α_2_δ-1ΔC in the presence of LRP1-m4

**Figure 6:**
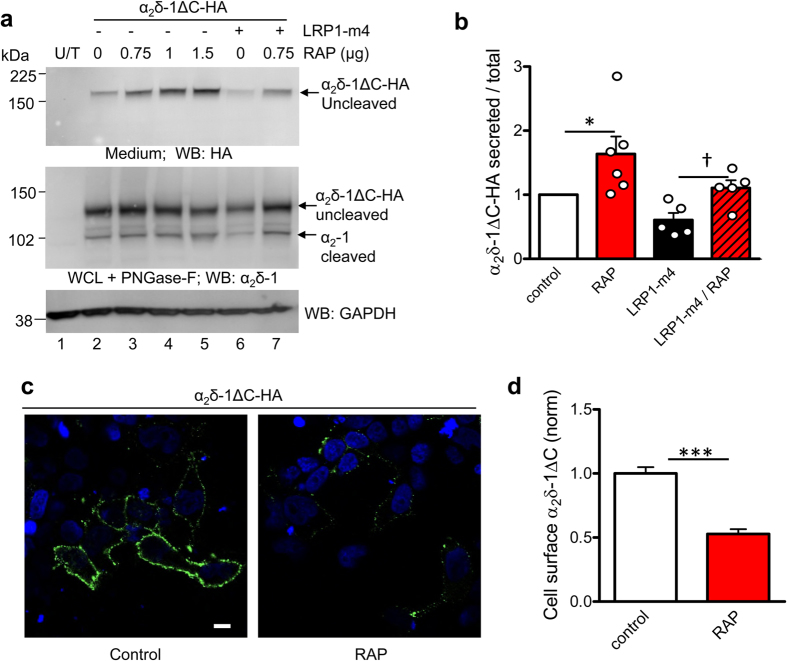
Effect of LRP1-m4 and RAP on secretion of anchorless α_2_δ-1ΔC. **(a)** α_2_δ-1ΔC-HA was expressed in tsA-201 cells either with the empty vector (lane 2) or with increasing concentrations of RAP (lanes 3–5), or with Flag-LRP1-m4 (lane 6) or Flag-LRP1-m4 plus RAP (lane 7). Lane 1 shows untransfected cells (U/T). Top panel: α_2_δ-1ΔC-HA detected in medium (HA Ab). Middle panel: α_2_δ-1ΔC-HA in WCL, deglycosylated with PNGase-F (α_2_δ-1 Ab). Note also the increase in cleaved α_2_-1 as RAP is increased (middle panel; lanes 2–5). Bottom panel: GAPDH loading control for WCL. **(b)** Quantification of experiments including that shown in (**b**), of α_2_δ-1ΔC-HA secreted into the medium, measured as ratio of WCL α_2_δ-1-ΔC, and normalised to control without RAP in each experiment (open bar, n = 6), for co-transfection with RAP (0.75 μg; red bar, n = 6), with Flag-LRP1-m4 alone (black bar, n = 5) or LRP1-m4 plus RAP (0.75 μg; red hatched bar, n = 5). Data are mean ± SEM with individual data points, *P = 0.0313, Wilcoxon’s matched pairs signed rank test compared to normalised control; ^†^*P* = 0.0234, paired t test. **(c)** Non-permeabilised tsA-201 cells, transfected with α_2_δ-1ΔC-HA, and cell surface-labelled with HA Ab, merged with DAPI staining. Left: control, transfected with α_2_δ-1ΔC-HA and empty vector; right: α_2_δ-1ΔC-HA co-transfected with 0.75 μg RAP. **(d)** Bar chart (mean ± SEM) showing effect of RAP (red bar, n = 150), normalised to control α_2_δ-1ΔC-HA cell surface expression (open bar, n = 187), for n = 3 independent experiments. ***P < 0.001, Student’s t test. Full blots for all figure parts are shown in Supplementary information.

Secretion of C-terminally truncated soluble LRP1 constructs has been used previously to study the role of the interaction of RAP with LRP1^47^. In order to determine whether membrane anchoring of α_2_δ-1 was required for its interaction with LRP1, we utilised the anchorless α_2_δ-1 construct described above (α_2_δ-1ΔC, cartoon in Fig. 3a), which can be secreted as a soluble protein^43^. We studied the effect of LRP1-m4 and the role of RAP on the secretion of α_2_δ-1ΔC into the medium. When we co-expressed α_2_δ-1ΔC with increasing concentrations of RAP, we found that secretion of α_2_δ-1ΔC was increased compared to the control in the absence of RAP (Fig. 6a, lanes 3–5 compared to lane 2, quantified in Fig. 6b). Furthermore, RAP also antagonised the inhibitory effect of LRP1-m4 on α_2_δ-1ΔC secretion (Fig. 6a, lane 7 compared to lane 6, quantified in Fig. 6b). This result shows that RAP antagonised the interaction of α_2_δ-1ΔC with both exogenous LRP1-m4 and also endogenous LRP1 (or related endogenous members of the LDL-receptor family), to promote secretion of α_2_δ-1ΔC.

We have previously found that while most is secreted, some α_2_δ-1ΔC remains associated with the cell surface of tsA-201 cells and DRG neurons by non-covalent interactions, and we speculated that another molecular interactor mediates this cell-surface association, in the absence of GPI-anchoring^43^. In the light of the interaction with LRP1 identified here, and the modulatory role of RAP in this interaction, we decided to investigate the effect of RAP on the cell surface association of α_2_δ-1ΔC. Co-expression of RAP with α_2_δ-1ΔC in tsA-201 cells strikingly reduced the amount of α_2_δ-1ΔC on the cell surface, as measured by cell surface immunofluorescence, compared to the control without RAP (Fig. 6c,d), without significantly affecting the total expression of α_2_δ-1ΔC measured in permeabilised cells (data not shown). This suggests that the attachment of α_2_δ-1ΔC might be mediated by an endogenous molecule belonging to the LDL-receptor family, to which α_2_δ-1ΔC remains associated through the trafficking pathway to the cell surface, such that the binding can be antagonised by RAP, allowing increased secretion of α_2_δ-1ΔC.

### LRP1 and RAP affect the N-glycosylation and proteolytic processing of α_2_δ-1

**Figure 7:**
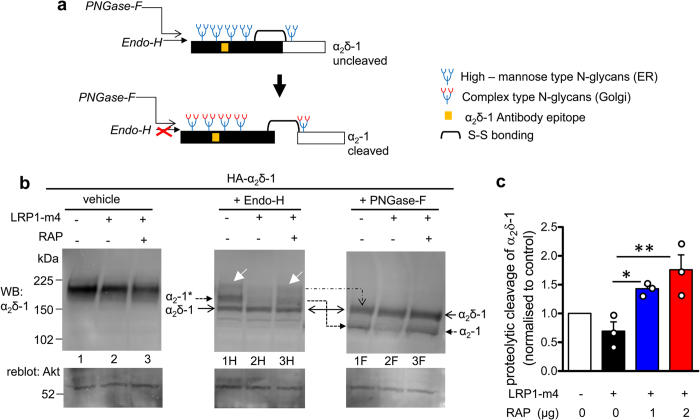
Effects of RAP and LRP1-m4 on α_2_δ-1 subunit trafficking impacts their glycosylation pattern and post-translational proteolytic cleavage. **(a)** Schematic representation of the different species of α_2_δ-1 N-glycosylation associated with proteolytic processing, indicating that the cleaved α_2_-1 is resistant to Endo-H treatment after its processing in the Golgi. **(b)** Western blot analysis: WCL from tsA-201 cells expressing HA-α_2_δ-1 either alone (lane 1), with Flag-LRP1-m4 (lane 2) or with Flag-LRP1-m4 and 1 μg RAP (lane 3). Top panel: α_2_δ-1 (α_2_-1 mAb), bottom panel, reblot for Akt loading control. Left panel shows glycosylated α_2_δ-1 (lanes 1–3), which does not resolve the different species of α_2_δ-1. Endo-H treatment (middle panel; lanes 1H-3H), targeting only the high–mannose (ER–associated), but not the complex (Golgi–associated) N-glycans, shows that cleaved α_2_-1 is resistant to Endo-H (α_2_-1* species; solid arrowhead on left), whereas uncleaved pro-α_2_δ-1 is sensitive to Endo-H (α_2_δ-1 species; open arrowhead on left). White arrows on blot shows a minor fraction of the uncleaved pro-α_2_δ-1, which is Endo-H resistant. Note that Endo-H-resistant (Golgi-associated) species of α_2_δ-1 are reduced upon co-expression of Flag-LRP1-m4 (middle panel; lane 2H), which is reversed by co-expression of RAP (middle panel; lane 3H). PNGase-F treatment (right panel; lanes 1F-3F) caused removal of all types of N-glycans from both cleaved α_2_-1 and uncleaved α_2_δ-1 (arrows on right). Note that cleavage of α_2_-1 is reduced upon co-expression of Flag-LRP1-m4, and this is reversed by co-expression of RAP (α_2_-1 species; compare lanes 2F and 3F). Representative of n = 2 experiments. **(c)** Quantification of α_2_δ-1 proteolytic processing expressed as a ratio of cleaved α_2_-1 to total α_2_δ-1 (cleaved + uncleaved) in PNGase-F deglycosylated WCL, normalised to control conditions (open bar), in the presence of LRP1-m4 (black bar) or LRP1-m4 plus 1 μg or 2 μg RAP cDNA (blue and red bars respectively). Data are mean ± SEM with individual data points for n = 3 independent experiments. **P < 0.01, *P < 0.05, 1-way ANOVA and Bonferroni’s post-hoc test. Full blots for all figure parts are shown in Supplementary information.

Next we examined whether LRP1-m4 and RAP would affect the N-glycosylation pattern and proteolytic cleavage of α_2_δ-1, in order to provide insight into where their interaction occurs. The α_2_δ-1 protein goes through a series of post-translational processing steps in the ER and Golgi, including N-glycosylation at multiple sites, and proteolytic cleavage of the pro-form of α_2_δ-1 into α_2_ and δ polypeptides (Fig. 7a)^48^. The core N-linked glycans, added co-translationally to the nascent proteins in the ER, are rich in mannose residues and termed high mannose-type or immature glycans. They are subsequently trimmed and modified in the Golgi to mature N-glycans^49^, although some membrane proteins by-pass this route^50^. The enzyme Endoglycosidase-H (Endo-H) discriminates between the two types of N-glycosylation, being able to strip the high mannose N-glycans from proteins, but unable to remove the mature N-glycans, formed by processing in the Golgi. The enzyme Peptide-N-Glycosidase-F (PNGase-F) is able to remove all types of N-glycosylation from proteins, without discrimination.

Since α_2_δ-1 is incompletely proteolytically cleaved when expressed in heterologous systems^43^, we investigated the N-glycosylation pattern of the cleaved α_2_-1 and uncleaved α_2_δ-1 proteins, as a marker of whether the mature cleaved α_2_-1 is derived from the ER or Golgi. We found that cleaved α_2_-1 is resistant to Endo-H (Fig. 7b; lane 1H, closed arrow) but sensitive to PNGase-F (Fig. 7b; lane 1F, closed arrow), showing that it contains mostly mature N-glycans, and suggesting that the proteolytic cleavage of α_2_δ-1 occurs after trimming of the glycans in the Golgi. In line with this result, the uncleaved pro-α_2_δ-1 form is mostly sensitive to both Endo-H (Fig. 7b; lane 1H, open arrow) and PNG-ase-F (Fig. 7b; lane 1F, open arrow), suggesting that this protein species derives mainly from ER, and has not yet been exported through the Golgi, where the N-glycans are modified.

Furthermore, co-expression of LRP1-m4 resulted in a reduction of mature Golgi-type N-glycosylation on α_2_-1 (Fig. 7b, compare lanes 1H and 2H), and reduced α_2_δ-1 proteolytic cleavage (Fig. 7b, compare lanes 1F and 2F, closed arrow), suggesting that it retards forward trafficking of α_2_δ-1. In contrast, when RAP was co-expressed with LRP1-m4, it antagonised this effect, and increased both mature N-linked glycosylation (Fig. 7b, lane 3H) and the cleavage of α_2_δ-1 (Fig. 7b; compare lanes 3F and 2F). Quantification of proteolytic cleavage of α_2_δ-1 showed that the additional presence of RAP increased α_2_δ-1 cleavage ~2-fold compared to LRP1-m4 alone (Fig. 7c). This is in agreement with the hypothesis that in the absence of RAP, an interaction between LRP1 and α_2_δ-1 early after their translation results in ER retention; this is antagonised by RAP, allowing forward trafficking. It is likely that RAP, by increasing the folding of LRP1, allows trafficking of both LRP1 and α_2_δ-1 to the Golgi. Our results suggest that proteolytic cleavage of α_2_δ-1 occurs in Golgi or post-Golgi compartments. It is also likely that α_2_δ-1 is trafficked in a complex with LRP1-m4, as we still detected interaction of LRP1-m4 with α_2_δ-1 on the cell surface (see Fig. 2c). This provides further evidence for the dual role of RAP in antagonising premature interaction between LRP1 and its ligands in the ER, as well as in trafficking of both LRP1 and its ligands.

### Effect of LRP1 and RAP on calcium currents and cell surface expression of Ca_V_2.2 and α_2_δ-1

**Figure 8:**
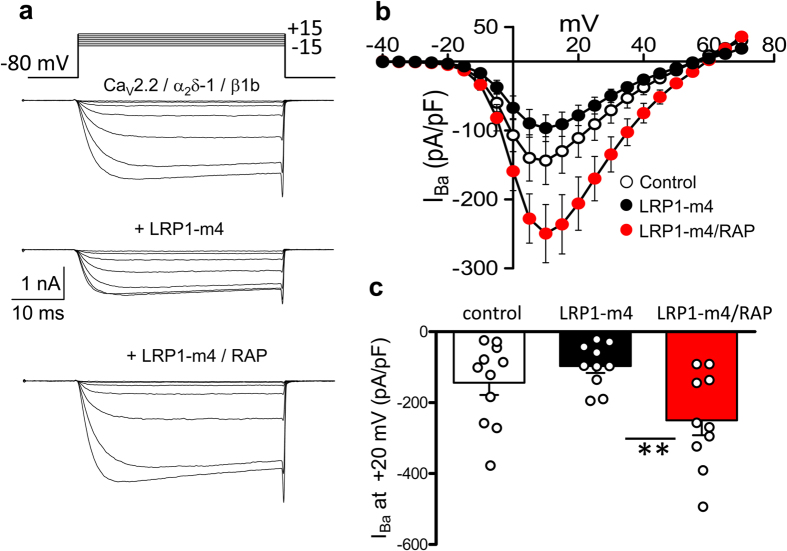
Co-expression of RAP with LRP1-m4 increases Ca_V_2.2 current density. **(a)** Example I_Ba_ current traces evoked by voltage steps from a holding potential of −80 mV to between −15 and +15 mV for tsA-201 cells transfected with Ca_V_2.2/β1b/α_2_δ-1 and control cDNA (top), LRP1-m4 (middle) or LRP1-m4/RAP (bottom). The charge carrier is 1 mM Ba^2+^. The scale bars refer to all traces. (**b**) Mean (±SEM) *IV* curves for control cells (open circles, n = 11), LRP1-m4-transfected cells (black circles, n = 10), or LRP1-m4/RAP-transfected cells (red circles, n = 10), from 3 independent experiments. **(c)** Comparison of peak I_Ba_ density at +10 mV (mean ± SEM with individual data points) for control cells (open bar), LRP1-m4-transfected cells (black bar) and LRP1-m4/RAP-transfected cells (red bar). **P < 0.01, 1-way ANOVA and Bonferroni’s post-hoc test.

We then examined whether the effects of LRP1-m4 and RAP on trafficking and proteolytic processing of α_2_δ-1 would translate into effects on calcium currents. Ca_V_2.2 was co-transfected with β1b and α_2_δ-1, with either a control cDNA, LRP1-m4 alone or LRP1-m4 plus RAP. The peak I_Ba_ current density in the presence of LRP1-m4 and RAP was 2.6-fold greater than in the absence of RAP, in agreement with the general hypothesis outlined above (Fig. 8a,b,c). Similar results were obtained using either an irrelevant protein vector or empty pcDNA3 vector in the control condition (data not shown).

**Figure 9:**
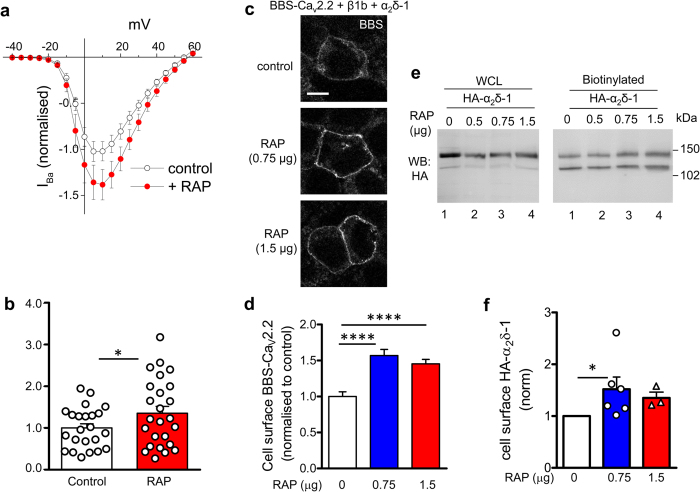
Expression of RAP alone enhances Ca_V_2.2 current density and Ca_V_2.2 cell surface expression. **(a)** Mean (±SEM) *IV* curves for I_Ba_ recorded from tsA-201 cells transfected with Ca_V_2.2/β1b/α_2_δ-1 (normalised to mean controls within each experiment), for control cells (open circles, n = 23) and cells transfected additionally with RAP (red circles, n = 25), from 2 independent experiments. The charge carrier is 1 mM Ba^2+^. **(b)** Comparison of normalised peak I_Ba_ density at +10 mV (mean ± SEM with individual data points) for control cells (open bar) and RAP-transfected cells (red bar). One data point was removed as an outlier (4.1-fold greater than the mean, P < 0.01 Grubb’s test). Statistical difference, Student’s t test: *P = 0.0193. **(c)** tsA-201 cells transfected with BBS-Ca_V_2.2/β1b/HA-α_2_δ-1 and cell-surface labelled with α-bungarotoxin AF-488. Top panel: control, transfected with empty vector; middle and bottom panels: co-transfected with 0.75 and 1.5 μg RAP, respectively. Scale bar 10 μm. **(d)** Bar chart (mean ± SEM) quantifying effect of the two RAP concentrations on cell surface BBS-Ca_V_2.2 signal (blue bar, 0.75 μg RAP, n = 246; red bar, 1.5 μg RAP, n = 285), normalised to control BBS-Ca_V_2.2 cell surface expression (open bar, n = 176), from three independent transfections, including that shown in (**c**). ****P < 0.0001, 1-way ANOVA and Bonferroni’s post-hoc test. **(e)** Example western blot from tsA-201 cells transfected with HA-α_2_δ-1, without RAP (lane 1) or with RAP (0.5, 0.75 and 1.5 μg, lanes 2–4), showing WCL (left panel) and corresponding cell surface biotinylation (right panel) for HA-α_2_δ-1. Ca_v_2.2 and β1b subunits were co-transfected for consistency with the experiments shown in parts (**a–d**) of this figure, but were not visualised by antibody staining. **(f)** Bar chart (mean ± SEM) and individual data points, for 0.75 μg (blue bar, ○, n = 6) and 1.5 μg RAP (red bar, ∆, n = 3)), showing effect of RAP on α_2_δ-1 cell surface expression determined by biotinylation from experiments including that in (**e**) normalised to control (open bar). *P = 0.0313, Wilcoxon’s matched pairs signed rank test. Combining all data, for both RAP concentrations, *P* < 0.0039 compared to control. Full blots for all figure parts are shown in Supplementary information.

Furthermore RAP alone produced a small increase in calcium currents, increasing the peak I_Ba_ by 36% (Fig. 9a,b), and increased the amount of Ca_V_2.2 on the cell surface, determined by α-bungarotoxin cell surface labelling of BBS-tagged Ca_V_2.2 by 56.9% for 0.75 μg RAP (Fig. 9c,d)^46^. Moreover, RAP alone also increased the cell surface expression of α_2_δ-1, determined by cell surface biotinylation, (Fig. 9e, compare lane 1 with lanes 3 and 4; quantified in Fig. 9f). The mean increase was 51.8% for 0.75 μg RAP. These effects of RAP are likely to be mediated either via an influence on endogenous LRP1 or another member of this family, and suggest that endogenous RAP may be limiting in these circumstances.

## Discussion

LRP proteins are widely recognised to be involved in trafficking and endocytosis of many protein ligands^21^,^51^, and loss of LRP1 in the central nervous system results in impaired synaptic function^29^,^30^. Furthermore, other LRP family members are also involved in a number of signalling pathways; for example LRP5/6 are co-receptors with the G-protein coupled receptor, frizzled, for Wnt signalling^22^. Here we show that the calcium channel auxiliary subunit α_2_δ-1 is a ligand for LRP1, which, when combined with its chaperone protein RAP, promotes the trafficking, cell surface expression, mature glycosylation and proteolytic cleavage of α_2_δ-1. We also provide evidence that LRP1 remains associated with α_2_δ-1 on the cell surface in the presence of RAP. The α_2_δ-1 subunit associates with Ca_V_1 and Ca_V_2 calcium channels, to promote their trafficking and voltage-dependent activation^6^. We find here that the LRP1/RAP combination also promotes functional expression of the neuronal (N-type) Ca_V_2.2 channels, in terms of both cell surface expression and increased calcium currents. We have recently shown that proteolytic processing of α_2_δ into α_2_ and δ is essential for this auxiliary subunit to enhance currents through Ca_V_2.2 channels^48^, and we find here that proteolytic cleavage of α_2_δ-1 is increased by LRP1/RAP, which is likely to contribute to their effect on calcium currents.

*CACNA2D1*, the gene encoding α_2_δ-1, is one of many genes whose expression is altered in sensory DRG neurons following peripheral nerve damage leading to neuropathic pain^15^,^52^,^53^. In contrast RAP is down-regulated in DRG neurons following sensory nerve injury^36^. The consequences of this for α_2_δ-1 trafficking and calcium channel function might be to retain some of the increased α_2_δ-1 in the ER because of premature interaction with LRP1. Relevant to this, we observed a substantial amount of α_2_δ-1 in the ER within rat DRG cell bodies following spinal nerve ligation^15^, and also observed uncleaved α_2_δ-1 in DRG somata^48^.

Of interest, the LRP1 gene has recently been identified as a susceptibility locus associated with common painful migraine^54^, and LRP1 has also been linked to the survival of Schwann cells and signalling in neuropathic pain^55^. Painful nerve injury also results in up-regulation of the extracellular matrix protein thrombospondin-4^56^. Thrombospondin family members are known LRP1 interacting proteins^32^. Indeed, we have previously confirmed this interaction, showing thrombospondin-4 to interact with the LRP1 mini-receptors used in the present study^40^. Thrombospondins have also been identified as binding partners for α_2_δ-1, by co-immunoprecipitation^19^. This interaction was found to be disrupted by gabapentin; and it was suggested that this might mediate a therapeutic action of gabapentin via inhibition of synaptogenesis^19^. In contrast, we found that there was no detectable interaction of the two proteins on the cell surface of transfected cells^40^. However the affinity of [^3^H]-gabapentin binding to α_2_δ-1 was reduced by co-expression of thrombospondin-4; therefore we concluded there could be a weak interaction between co-expressed α_2_δ-1 and thrombospondin-4 in membranes derived from intracellular compartments. In the present study we have shown that LRP1 interacts with α_2_δ-1, both by immunoprecipitation and by pull-down with purified LRP1 mini-receptors. Therefore it is possible, since LRP1 has multiple LBDs, that LRP1 (or a related family member) could contribute to bridging an indirect interaction between α_2_δ-1 and thrombospondins, or indeed between PrP and α_2_δ-1^57^.

Although LRP1 is not primarily a DRM-associated protein, it has previously been observed in DRMs from some cell types^58^. Furthermore, it does interact with some DRM-associated proteins, for example with PrP^25^, which has been found to result in signalling in specific DRM domains^58^,^59^. LRP1 also affects PrP endocytosis in a RAP-dependent manner^25^. We find here that a small proportion of LRP1 is present in mouse brain DRMs, and here it co-immunoprecipitates a comparable fraction of α_2_δ-1 and PrP, both being established DRM residents. The relatively small co-immunoprecipitated fraction relative to the total α_2_δ-1 and PrP in DRMs is in line with the partial partitioning of LRP1 into DRMs.

LRP1 is known to be recruited to caveolae^59^, and to be involved in endocytosis^60^. Whether LRP1 and RAP also influence α_2_δ-1 endocytosis, in the same way as for PrP, and whether the same LRP1 molecule can bind both α_2_δ-1 and PrP ligands occupying different sites, remains to be determined in future studies.

LRP1 binds multiple ligands^37^, including von Willebrand factor^61^, and this contributes to the clearance of von Willebrand factor which is internalised from blood plasma by macrophages^62^. LRP1 also binds to the inserted VWA domain in α_M_β_2_ integrin^51^. A poly-basic motif at the start of the VWA domain is implicated in this binding, rather than a direct interaction with the VWA domain MIDAS motif. It is of interest that one of the key motifs mediating gabapentin binding to α_2_δ-1 and α_2_δ-2 is a basic triple arginine sequence, just prior to its VWA domain^8^,^12^,^63^. In the present study we found that the number of gabapentin binding sites (B_max_) associated with α_2_δ-1 was markedly reduced by co-expression of LRP1. It is thus possible that LRP1 binding to α_2_δ-1 involves the gabapentin binding pocket motif, thus occluding the gabapentin binding site.

Despite our evidence that endogenous LRP1 is a good candidate for interaction with α_2_δ-1, both during trafficking and on the cell surface, at this point we cannot exclude other endogenous members of LDL–receptor family of proteins, such as LRP5/6 which also interact with RAP^64^, and are involved in Wnt signalling^22^. It will be of interest in the future to determine whether these LRP family members interact with α_2_δ proteins.

## Methods

### Molecular biology

The cDNAs used were: rat α_2_δ-1 (M86621), rabbit Ca_V_2.2 (D14157 without 3′ UTR), and rat β1b (X61394). In some experiments Ca_V_2.2 was used with an extracellular BBS tag (BBS-Ca_V_2.2)^46^. Other α_2_δ-1 constructs used were α_2_δ-1-ΔC-HA (C-terminal HA tag)^43^ and HA-α_2_δ-1 (HA-tag sequence YPYDVPDYA inserted between Asn-549 and Asp-550)^43^. These cDNAs were in the pMT2 and pCDNA3 vectors for expression in tsA-201 cells. LRP1 mini-receptors m1-m4 cDNAs in pcDNA3^39^ and RAP pcDNA3 were gifts from Dr. Guojun Bu (Washington University, St Louis, USA). A triple Flag tag (22 amino acids) was inserted in place of the original HA tag in the LRP1 mini-receptor constructs, between amino acids 24 and 25^40^. Other cDNAs used were β1b-GFP^65^, P2 × 2^K69A^ (gift from Prof. R. A. North, Manchester University), Kir2.1^AAA^ (gift from Prof. A Tinker, William Harvey Research Institute, Queen Mary University of London) and CD8 transfection marker.

### Antibodies and other materials

The α_2_δ-1 antibody used is a mouse monoclonal against the α_2_-1 moiety (Sigma-Aldrich); the epitope is identified in ref. 46. Other antibodies used were anti-HA (rat monoclonal, Roche, used for immunocytochemistry), anti-HA (rabbit polyclonal, Sigma, used for most western blots), anti-GAPDH (mouse monoclonal, Ambion), anti-flotillin-1 (mouse monoclonal, BD Biosciences), anti-Akt/PKB (rabbit polyclonal, Cell Signaling Technologies); anti-M2 Flag (Sigma), polyclonal anti-LRP1 antibody raised against purified LRP1, which also recognises RAP (gift and personal communication from Dr. S. Moestrup, Department of Biomedicine, Aarhus University, Denmark) and anti-PrP (3F4 epitope)^57^ (gift from Dr. Roberto Chiesa). For immunocytochemistry, secondary Abs (1:500) used were anti-rat-Alexa Fluor 594 or anti-rat-fluorescein isothiocyanate (FITC) (Sigma-Aldrich).

### Cell culture and transfection

The tsA-201 cells (European Collection of Authenticated Cell Cultures), tested to be mycoplasma-free, were cultured in Dulbecco’s modified Eagle’s medium (DMEM) supplemented with 10% foetal bovine serum (FBS), 1 unit/ml penicillin, 1 μg/ml streptomycin and 1% GlutaMAX (Life Technologies, Waltham, MA). They were plated onto cell culture flasks, coverslips or glass-bottomed dishes (MatTek Corporation, Ashland, MA), coated with poly-L-lysine, and cultured in a 5% CO_2_ incubator at 37 °C. Cells were transfected using Fugene6 (Promega, Fitchburg, WI), according to the manufacturer’s protocol. For biochemical experiments cells were harvested 48 h after transfection, by washing cells in phosphate-buffered saline (PBS, pH 7.4, Sigma), detaching with a cell scraper into PBS, and pelleting by centrifugation at 1,000 × g.

For electrophysiology the DNA ratios for Ca_V_2.2/β1b/α_2_δ-1 were maintained constant within each set of experiments, usually at 3:2:2, and either empty vector or a non-functional membrane protein P2 × 2^K69A^ or Kir2.1^AAA^ were used in controls in place of LRP1-m4 or RAP, in order to maintain constant the total amount of cDNA transfected In some experiments β1b-GFP was used in place of β1b, to detect transfected cells. Site-directed mutagenesis was carried out using standard procedures, and all subcloning and mutations confirmed by sequencing.

### Transgenic mice

Conventional α_2_δ-1 knockout (α_2_δ-1^−/−^) mice on C57Bl/6 background were used^66^,^67^,^68^. Mice were housed in groups of no more than 5 on a 12 h:12 h light dark cycle; food and water were available *ad libitum.* Brains were obtained from male 10-week old α_2_δ-1^−/−^ mice and α_2_δ-1^+/+^ littermates, obtained by breeding from heterozygotes, during the course of a previous study^67^, and frozen at −80 °C until use. All experimental methods, procedures and protocols relating to breeding and obtaining tissue from these mice were approved by the UK Home Office and by the UCL local ethical committee. All methods were performed in accordance with the relevant guidelines and regulations.

### Western blotting

Sodium dodecyl sulfate (SDS)-polyacrylamide gel electrophoresis (PAGE) was carried out as previously described^43^. The following secondary Abs were used for western blotting: goat anti-rabbit, goat anti-rat and goat anti-mouse Abs coupled to horseradish peroxidase (HRP) (Biorad). The signal was obtained by HRP reaction with fluorescent product (ECL 2; Thermo Scientific), and membranes were scanned on a Typhoon 9410 phosphorimager (GE Healthcare). Full blots for all figures are shown in Supplementary information.

### Preparation of Triton X-100-insoluble DRMs and co-immunoprecipitation

The protocol was similar to that described previously^7^,^43^. All steps were performed on ice. Whole brains (without cerebellum) from WT and α_2_δ-1^−/−^ mice were used as the starting material. Tissue was homogenised using a Teflon homogeniser in Mes-buffered saline (MBS), containing 25 mM Mes (pH 6.5), 150 mM NaCl and protease inhibitors (PI, cOmplete, Roche; used according to manufacturer’s instructions), containing 1% (v/v) Triton X-100 (Thermo Scientific), and left on ice for 1 h. The sample was then supplemented with 90% sucrose to 45% final concentration and overlaid with 10 ml of discontinuous sucrose gradient, consisting of 35% (w/v) sucrose in MBS (5 ml) and 5% (w/v) sucrose in MBS (5 ml), centrifuged at 138,000 × g for 18 h at 4 °C (Beckman SW40 rotor). Fractions (1 ml) were subsequently harvested from the top to the bottom of the tube, and DRM fractions from the gradient were washed free of sucrose by dilution in 25 volumes of ice-cold PBS, and ultracentrifugation (150,000 × g for 1 h at 4 °C) to pellet the DRMs to be used for co-immunoprecipitation as described below.

### Cell surface biotinylation, cell lysis, deglycosylation and immunoblotting

The procedures were modified from those described previously^7^. Briefly, 48 h after transfection, tsA-201 cells were incubated for 30 min at room temperature with 0.5 mg/ml EZ-link Sulfo-NHS-LC-Biotin (Thermo Scientific) in PBS, and the reaction was quenched with 200 mM glycine. The cells were lysed for 45 min on ice in PBS, containing 1% Igepal; 0.1% SDS and PI. The WCL was then centrifuged at 20,000 × g at 4 °C for 20 min, and the pellet discarded. The supernatant was assayed for total protein (Bradford assay, Biorad). Immunoblot analysis was performed essentially as described previously^43^. Cleared WCL corresponding to 20–40 μg total protein was diluted with Laemmli sample buffer^7^, supplemented with 100 mM dithiothreitol (DTT), incubated at 60 °C for 10 min and resolved by SDS-PAGE and western blotting. Biotinylated lysates (adjusted to between 0.5 and 1 mg/ml total protein concentration) were applied to 40 μl prewashed streptavidin-agarose beads (Thermo Scientific) and rotated overnight at 4 °C. The beads were then washed 3 times with PBS containing 0.1% Igepal and, when required, the streptavidin beads were re-suspended in PNGase-F buffer (containing PBS, supplemented with 75 mM β-mercaptoethanol, 1% Igepal, 0.1% SDS, and PI) and deglycosylated for 3 h at 37 °C with PNGase-F (Roche Applied Science). When required, WCL were deglycosylated with PNGase-F under identical conditions described above or with Endo-H (NEB) following the manufacturer’s instructions. The samples were then resuspended in an equal volume of 2 × Laemmli buffer with 100 mM DTT, followed by 10 min incubation at 60 °C, prior to analysis by SDS-PAGE and western blotting.

### Co-immunoprecipitation

The assay was done as previously described with modifications^48^. Cell pellets were resuspended in co-immunoprecipitation buffer (50 mM Tris, pH 7.4, 150 mM NaCl, 1% Igepal, 0.5% Na deoxycholate, 0.1% SDS, 1 mM MgCl_2_, 1 mM CaCl_2,_ supplemented with EDTA-free PI), homogenised by 5 × passes through a 23 gauge syringe and 5 s sonication at 20,000 Hz, and then incubated for 1 h on ice. Insoluble material was removed by centrifugation at 20,000 × g for 30 min at 4 °C. The supernatant was incubated with 2 μg/ml of anti-M2-Flag antibody overnight at 4 °C with constant rotation. 30 μl prewashed A/G PLUS Agarose beads (Santa Cruz) were added to each tube and further rotated for 2 h at 4 °C. The beads were then pelleted by 500 × g centrifugation at 4 °C and washed twice with a co-immunoprecipitation wash high detergent buffer (1% Igepal, 20 mM Tris pH 7.4, 150 mM NaCl, 1 mM MgCl_2_, 1 mM CaCl_2,_ with EDTA-free PI), twice with co-immunoprecipitation wash high salt buffer (0.1% Igepal, 20 mM Tris pH 7.4, 500 mM NaCl, with EDTA-free PI), and twice with a low salt co-immunoprecipitation wash (0.1% Igepal, 20 mM Tris pH 7.4, with EDTA-free PI). Native LRP1 and α_2_δ were precipitated from whole brain DRMs concentrated as described above. DRMs were resuspended in a buffer consisting of 20 mM Tris pH 7.4, 150 mM NaCl, 1% 1-O-Octyl-β-D-glucopyranoside (OG; Sigma) and PI; then sonicated for 10 s at 20,000 Hz, rotated at 4 °C for 1 h and centrifuged at 20,000 × g at 4 °C. The supernatants were assayed for total protein, concentrations were equalised, and 20 μg/ml of rabbit anti-LRP1 or 20 μg/ml of rabbit non-specific IgG were added to the samples and rotated overnight at 4 °C. The samples were washed four times in the same buffer, but supplemented with 0.2% OG. The beads, with co-immunoprecipitated proteins, were resuspended in an equal volume of 2 × Laemmli buffer with 100 mM DTT, and analysed by SDS-PAGE and western blotting, together with aliquots of the initial WCL prior to co-immunoprecipitation.

### Cell surface co-immunoprecipitation

The protocol was performed as described previously^25^, with modifications. 48 h after transfection, tsA-201 cells were rinsed twice with PBS and incubated with gentle rocking at 4 °C for 45 min with 4 μg/ml monoclonal anti-M2-Flag antibody, diluted in a cold serum-free DMEM, supplemented with 10 mM HEPES pH 7.4. Cells were harvested and all subsequent procedures were performed on ice. The cell pellet was resuspended in co-immunoprecipitation buffer (50 mM Tris, pH 7.4, 150 mM NaCl, 1% Igepal, 0.5% Na deoxycholate, 0.1% SDS, 1 mM MgCl_2_, 1 mM CaCl_2_, with EDTA-free PI), homogenised as in co-immunoprecipitation, and incubated for 1 h on ice followed by centrifugation at 1000 × g for 5 min at 4 °C. The supernatants were collected and assayed for total protein (Bradford assay) and the concentration was adjusted to 1 mg/ml with co-immunoprecipitation buffer. 40 μl pre-washed A/G PLUS-Agarose beads (Santa Cruz) were added to each sample followed by rotation for 1 h at 4 °C. The beads were then pelleted by centrifugation (500 × g; 4 °C), washed twice with co-immunoprecipitation wash buffer (20 mM Tris, pH 7.4, 150 mM NaCl, 0.5% Igepal, 1 mM MgCl_2_, 1 mM CaCl_2_, with PI) and the sample was resupended in an equal volume of 2 × Laemmli buffer with 100 mM DTT, and analysed by SDS-PAGE and western blotting.

### Affinity purification of secreted α_2_δ-1ΔC-HA from conditioned media

tsA-201 cells were transfected with α_2_δ-1ΔC-HA and incubated for 48 h in a 5% CO_2_ incubator at 37 °C in DMEM medium supplemented with 2% FBS, 1 unit/ml penicillin, 1 μg/ml streptomycin and 1% GlutaMAX. The conditioned medium from 6 flasks was pooled, filtered (0.2 μm membrane pore size) and concentrated (x 40 fold) on a 10 kDa Nominal Molecular Weight Limit (NMWL) Amicon column (Merck Millipore) following the manufacturer’s instructions. The concentrated material was supplemented with 20 mM HEPES pH 7.4 and OG (Sigma) to 0.2% final concentration, and applied to a 1 ml agarose-anti-HA antibody column (Sigma), pre-equilibrated in wash buffer (20 mM HEPES pH 7.4, 200 mM NaCl, 0.2% OG), and rotated for 1 h at 20 °C. The column was washed extensively in wash buffer, and bound proteins were then eluted with 3 ml 100 mM glycine pH 2.5 in 0.5 ml fractions into tubes containing 50 μl 1 M HEPES pH 7.4 for immediate neutralisation. The eluates were applied to 3 ml Slide-A-Lyzer™ Dialysis Cassettes (3.5 K MWCO, Thermo Fisher Scientific) and dialysed overnight at 4 °C against 2 L of PBS, pH 7.4. Then aliquots of purified protein were analysed by SDS-PAGE and western blotting with anti-HA antibody, and on a silver stained gel, on which the concentration was estimated by running BSA standards of known concentration (not shown).

### Dot blot binding assay

The assay was a modification of that described previously^44^. 0.2 μg of purified Ligand binding cluster-II of LRP1 Recombinant Human protein (LRP1-m2 LBD; Thermo Fisher Scientific; dissolved to 0.1 mg/ml) or 0.2 μg BSA as negative control were immobilised on dry nitrocellulose membranes (Biorad). Membranes were then blocked for 1 h at 20 °C with dot blot buffer (PBS supplemented with 0.5% Igepal, 3% BSA, 1 mM MgCl_2_, and 1 mM CaCl_2_). Purified α_2_δ-1ΔC-HA was then diluted to 50 nM in dot blot buffer, and applied to the membranes for 1 h at 20 °C with gentle rocking. This was followed by three washes with dot blot wash buffer (dot blot buffer without BSA), and incubated for 1 h at 20 °C with anti-HA antibody diluted 1:1000 in dot blot buffer, three additional washes with dot blot wash buffer, and 30 min incubation at 20 °C with anti-rabbit-HRP diluted 1:3000 in dot blot buffer. After three final washes with dot blot wash buffer and a final wash with dot blot wash buffer without detergent, the proteins with bound antibodies were visualised with ECL 2.

### *In vitro* binding technique

tsA-201 cells were either not transfected or transfected separately with cDNA encoding HA-α_2_δ-1 or Flag-LRP1-m4/RAP (cDNA ratio 10:1). Cell pellets were homogenised at 4 °C in binding buffer (PBS supplemented with 25 mM sucrose, 1.5 mM CaCl_2_, 1 mM MgCl_2_, EDTA-free PI, and 0.5% Igepal) as for co-immunoprecipitation, and incubated on ice for 1 h. The lysates were cleared by 20,000 × g centrifugation at 4 °C and supernatants were collected and assayed for total protein (Bradford assay). The lysates from Flag-LRP1-m4/RAP and untransfected cells were rotated overnight at 4 °C with 10 μg/ml anti-M2-Flag antibody, then 50 μl of beads (prewashed in binding buffer) were added, and the sample was rotated for 2 h at 4 °C followed by 3 × washes with wash buffer (binding buffer supplemented with 0.1% Igepal). HA-α_2_δ-1 lysate was then equilibrated to 20 °C, and applied to the beads, and the samples were incubated for 2 h at 20 °C followed by 3 × washes with wash buffer and elution by heating in 2 × Laemmli buffer with 100 mM DTT prior to analysis by SDS-PAGE and western blotting.

### Immunocytochemistry, imaging and analysis

The procedure in tsA-201 cells was performed essentially as described previously with minor modifications^7^,^43^. Briefly, 48 h post-transfection the cells were fixed with 4% paraformaldehyde (PFA) in PBS at 20 °C for 5 min, and then incubated in PBS for 15 min. Blocking was performed for 1 h at 20 °C in PBS containing 20% goat serum and 5% bovine serum albumen (BSA). The primary antibody was then applied (diluted in PBS with10% goat serum and 2.5% BSA) overnight at 4 °C. The secondary antibody was applied (1:500 dilution in PBS, containing 2.5% BSA and 10% goat serum) at 20 °C for 1 h. In live-labelling experiments, cells expressing BBS-Ca_V_2.2 together with α_2_δ-1 and β1b were washed with Krebs Ringer HEPES (KRH) buffer, labelled with α-bungarotoxin (BTX)-AF 488 (Invitrogen; 1:100 in KRH buffer) at 17 °C for 30 min, then washed with KRH and fixed as described above^46^. Cell nuclei were stained with 0.5 μM 4′,6′-diamidino-2-phenylindole (DAPI) in PBS for 5 min. The coverslips were mounted onto glass slides using VECTASHIELD^®^ mounting medium (Vector Laboratories, Peterborough, UK).

Imaging was performed on a Zeiss LSM 780 confocal microscope as described elsewhere^46^. Images were obtained at fixed microscope settings for all experimental conditions of each experiment. Images (1024 × 1024 pixels) of tsA-201 cells were obtained using a 40× or 63× objective and an optical section of 0.8–1 μm. Every cell identified as transfected was included in the measurements, to ensure lack of bias. Images were analysed using imageJ (imagej.net) using a modification of the procedure described previously^7^,^43^. Surface labelling in non-permeabilised cells was measured using the freehand line tool and manually tracing the surface of the cell. The value of the mean pixel intensity in different channels was measured separately and background was subtracted by measuring the intensity of an imaged area without transfected cells. All data were then normalised to the appropriate positive control for each experiment before combining experiments.

### Electrophysiology

Calcium channel currents in transfected tsA-201 cells were investigated by whole cell patch clamp recording, essentially as described previously^48^. Cells were selected either using co-transfection with CD8 antigen, or by using β1b-GFP^65^, with essentially equivalent results, which were combined. The patch pipette solution contained in mM: Cs-aspartate, 140; EGTA, 5; MgCl_2_, 2; CaCl_2_, 0.1; K_2_ATP, 2; Hepes, 10; pH 7.2, 310 mOsm with sucrose. The external solution for recording Ba^2+^ currents contained in mM: tetraethylammonium (TEA) Br, 160; KCl, 3; NaHCO_3_, 1.0; MgCl_2_, 1.0; Hepes, 10; glucose, 4; BaCl_2_, 1, pH 7.4, 320 mosM with sucrose. Unless otherwise stated, 1 mM extracellular Ba^2+^ was the charge carrier. An Axopatch 1D or Axon 200B amplifier was used, with pipettes of resistance 2–4 MΩ. Whole cell voltage-clamp recordings were sampled at 10 kHz frequency, filtered at 2 kHz and digitized at 1 kHz. 70–80% series resistance compensation was applied and all recorded currents were leak subtracted using P/8 protocol. Membrane potential was held at −80 mV. Analysis was performed using Pclamp 9 (Molecular Devices) and Origin 10 (Microcal Origin, Northampton, MA).

### [^3^H] gabapentin binding assay

Binding of [^3^H]-gabapentin to membranes from tsA-201 cells was carried out essentially as previously described^40^, in a final volume of 250 μl at room temperature for 45 min. The cDNA for Kir2.1^AAA^ was used in control transfections to maintain the amount of total cDNA constant, and in order to have cells expressing similar amounts of protein under the experimental conditions used. Western blots for α_2_δ-1 expression were quantified for every experiment to ensure similar expression levels. Membrane fractions (60 μg of protein per tube) were incubated with various concentrations of [^3^H]-gabapentin (specific activity 36 Ci/mmol, American Radiolabeled Chemicals, St. Louis, MO, USA) in 10 mM HEPES/KOH pH 7.4, then rapidly filtered through GF/B filters, pre-soaked with 0.3% polyethyleneimine. Filters were washed three times with 3 ml ice-cold 50 mM Tris/HCl, pH 7.4 and counted on a scintillation counter. Concentrations of [^3^H]-gabapentin greater than 50 nM were achieved by adding non-radioactive gabapentin and correcting the specific binding by the dilution factor^4^. Non-specific binding was determined in the presence of 20 μM non-radioactive gabapentin. Data points were determined in triplicate, and data were analysed by fitting specific binding to the Hill equation^40^.

### Data analysis

Data are given as mean ± SEM, with individual data points if <30. Statistical comparisons were performed using either Student’s t test, paired t test, Wilcoxon’s matched pairs signed rank test, ANOVA with appropriate post-hoc test, as stated, using Graphpad Prism 5.

## Additional Information

**How to cite this article**: Kadurin, I. *et al*. LRP1 influences trafficking of N-type calcium channels via interaction with the auxiliary α_2_δ-1 subunit. *Sci. Rep.* **7**, 43802; doi: 10.1038/srep43802 (2017).

**Publisher's note:** Springer Nature remains neutral with regard to jurisdictional claims in published maps and institutional affiliations.

## Supplementary Material

Supplementary Information
