# Supplementary material for: LRP1 influences trafficking of N-type calcium channels via interaction with the auxiliary α2δ-1 subunit
Source: Sci Rep. 2017 Mar 3;7:43802. doi: 10.1038/srep43802 (PMC5335561; doi:10.1038/srep43802)

LRP1 influences trafficking of N-type calcium channels via interaction with the auxiliary $\alpha_2\delta$-1 subunit

Ivan Kadurin, Simon W Rothwell, Beatrice Lana, Manuela Nieto-Rostro and Annette C Dolphin

Supplementary Information

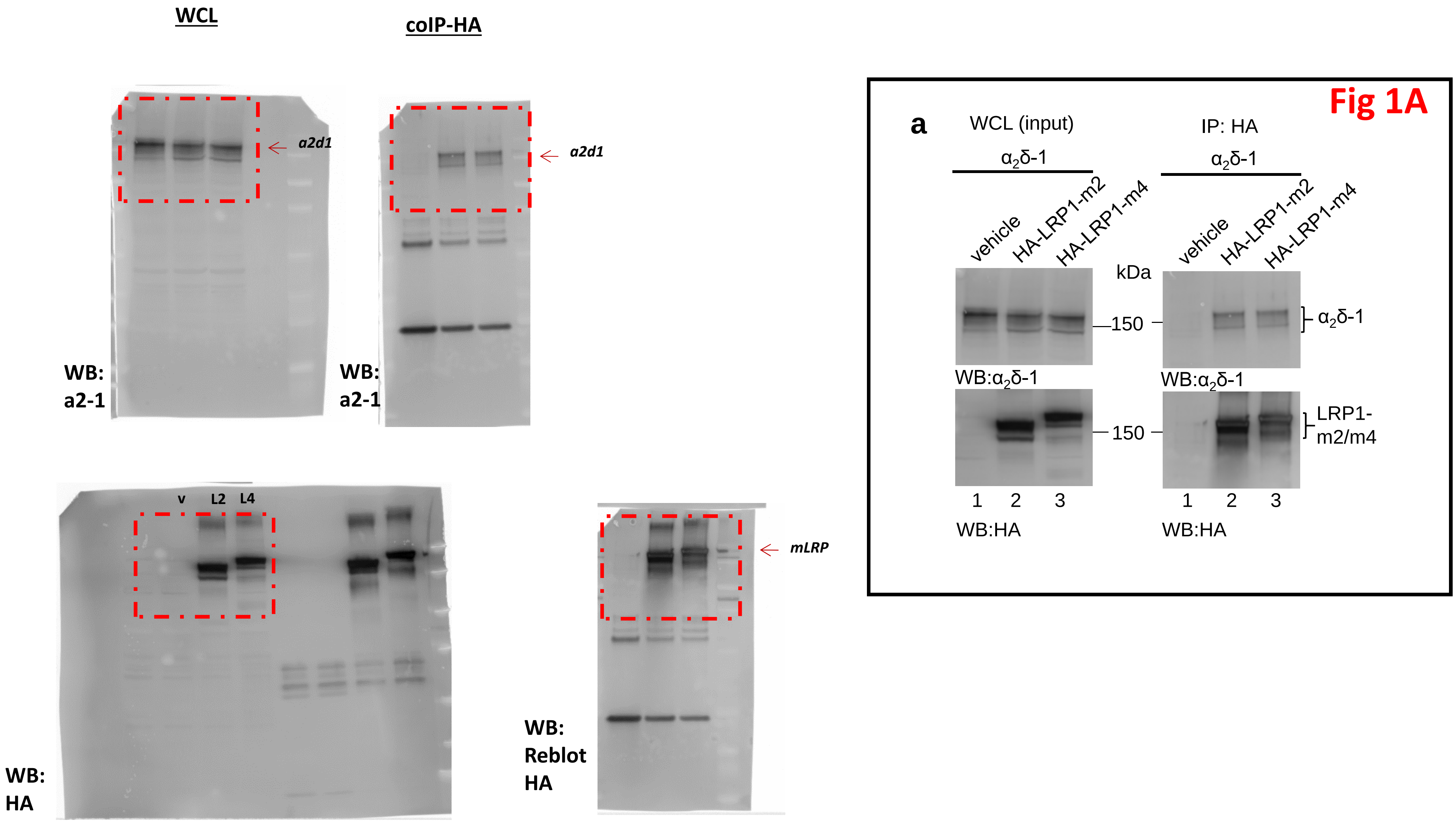
WCL
coIP-HA
a2d1
a2d1
WB: a2-1
WB: a2-1
v
L2
L4
WB: HA
mLRP
WB: Reblot HA
Fig 1A
a
WCL (input)
IP: HA
$\alpha_2\delta$-1
$\alpha_2\delta$-1
vehicle
HA-LRP1-m2
HA-LRP1-m4
vehicle
HA-LRP1-m2
HA-LRP1-m4
kDa
150
150
$\alpha_2\delta$-1
WB:$\alpha_2\delta$-1
WB:$\alpha_2\delta$-1
LRP1-m2/m4
1 2 3
1 2 3
WB:HA
WB:HA

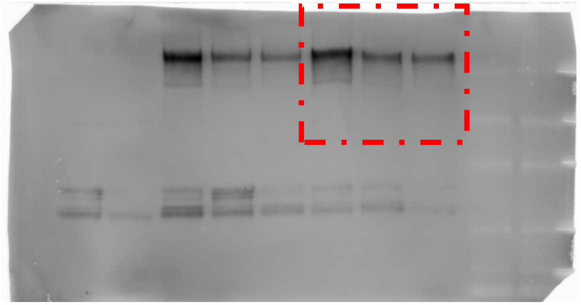

WB: HA

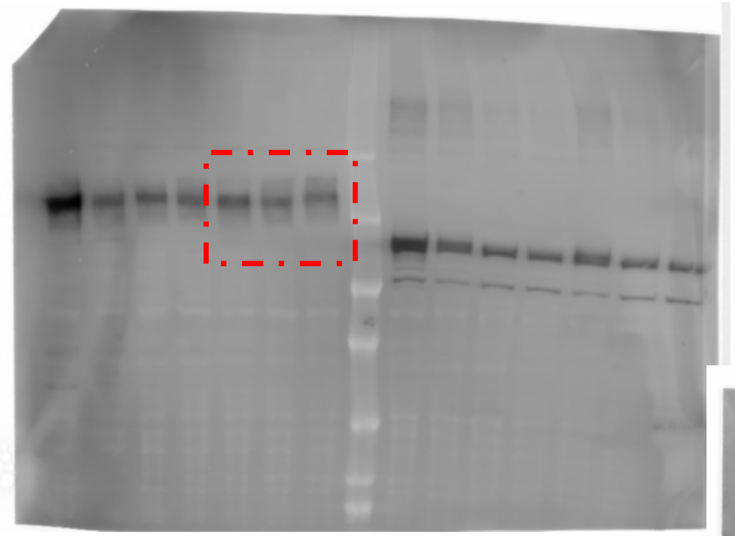

WB: HA

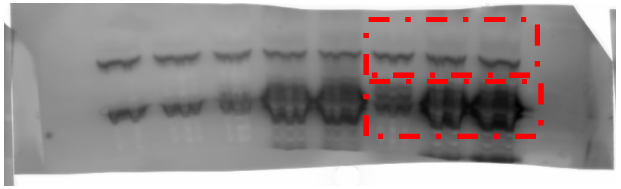

WB: Akt and RAP reblot

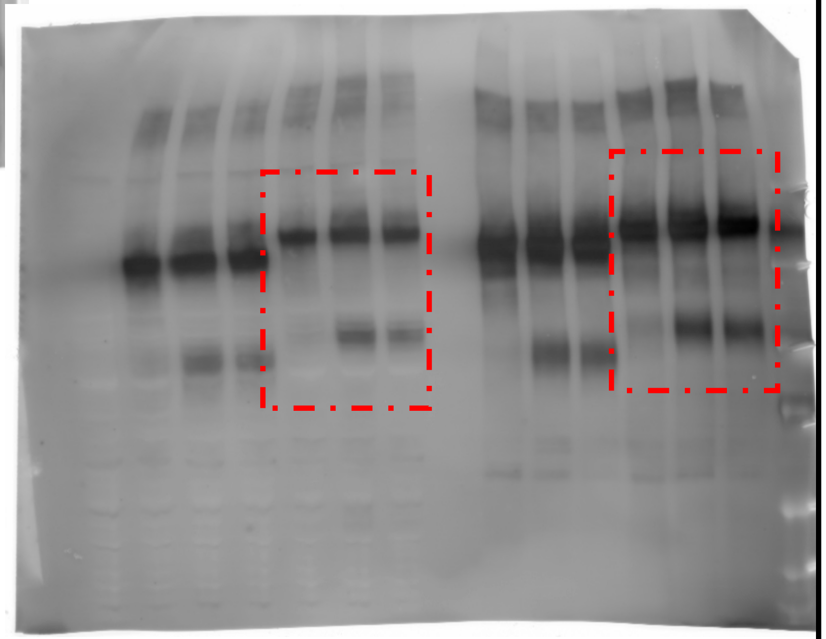

WB: FLAG

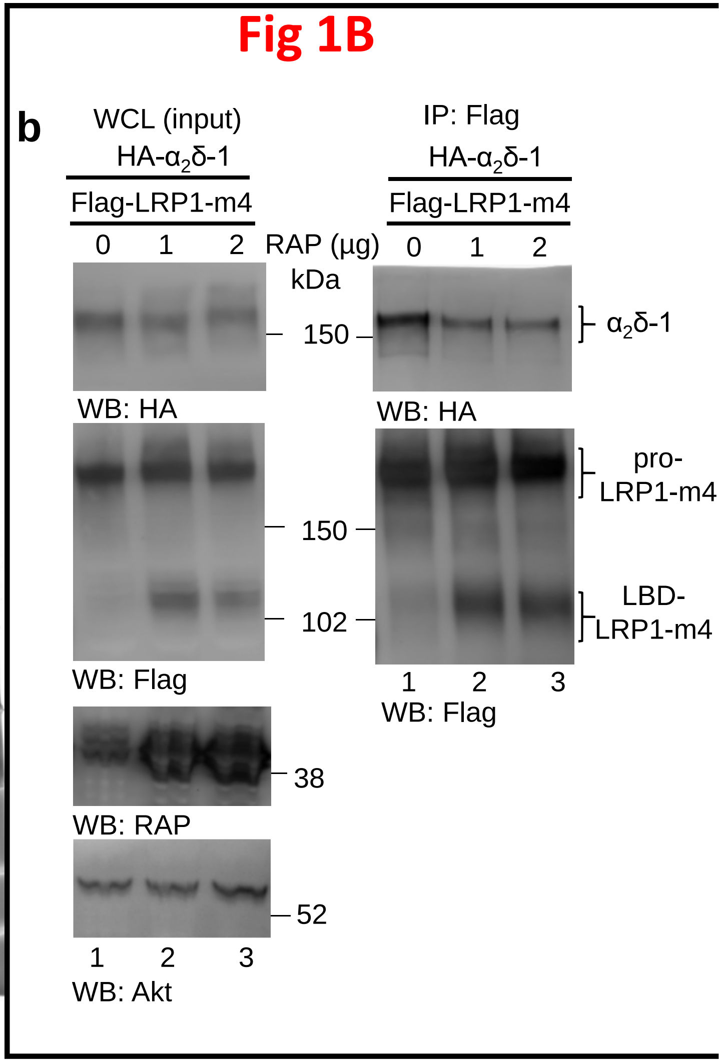

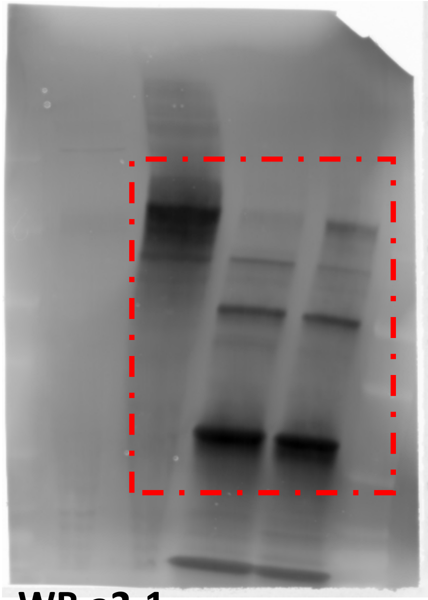

WB a2-1

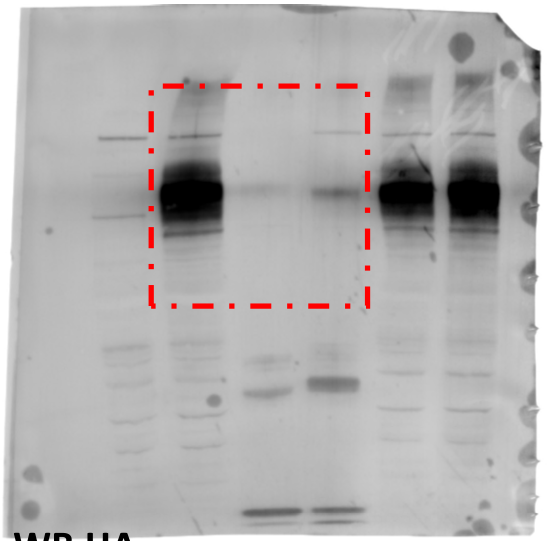

WB HA

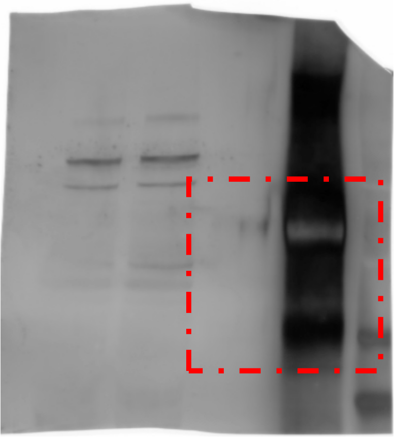

WB: FLAG

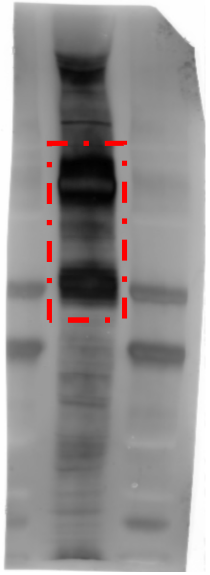

WB: FLAG

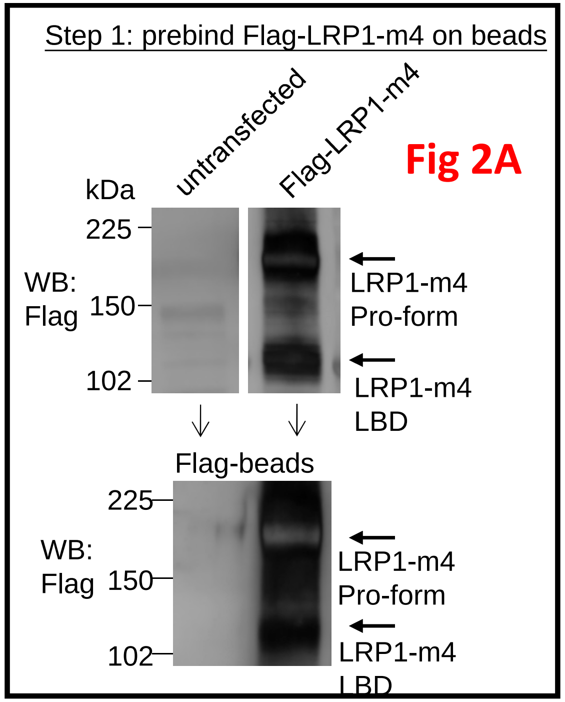

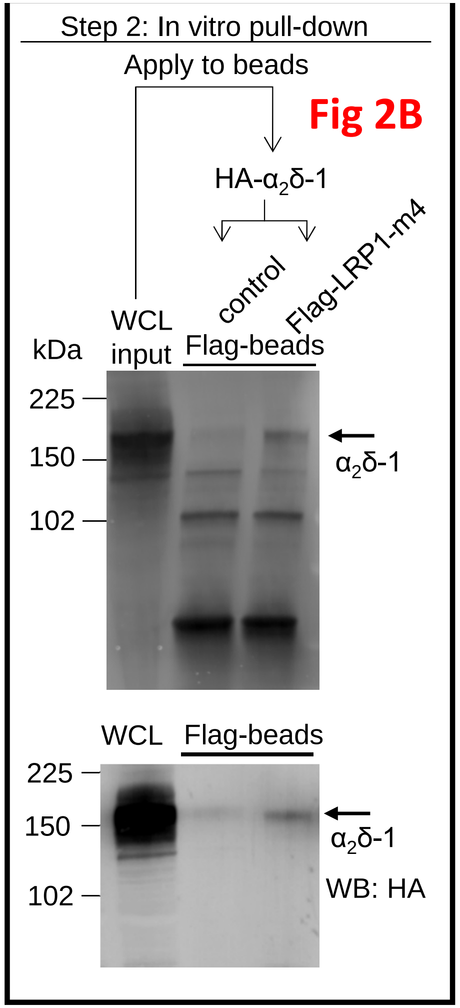

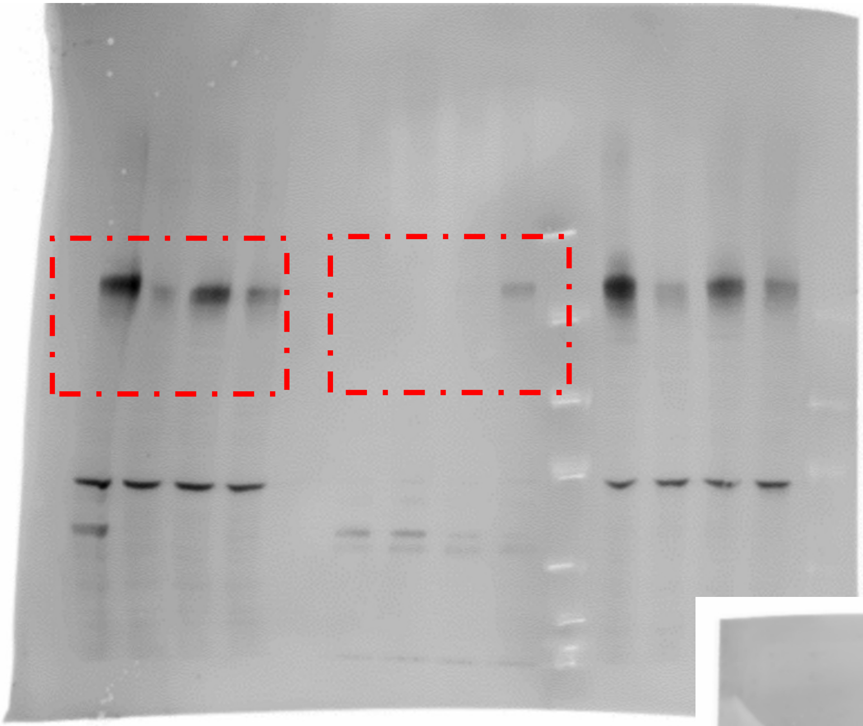

WB: a2-1

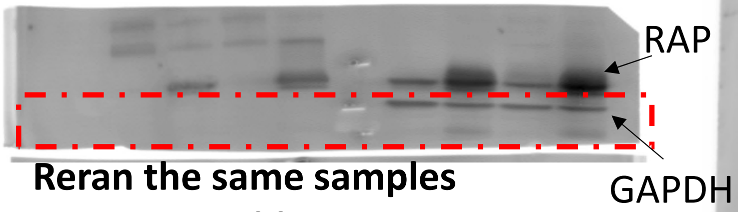

Reran the same samples
WB: RAP; Reblot: GAPDH

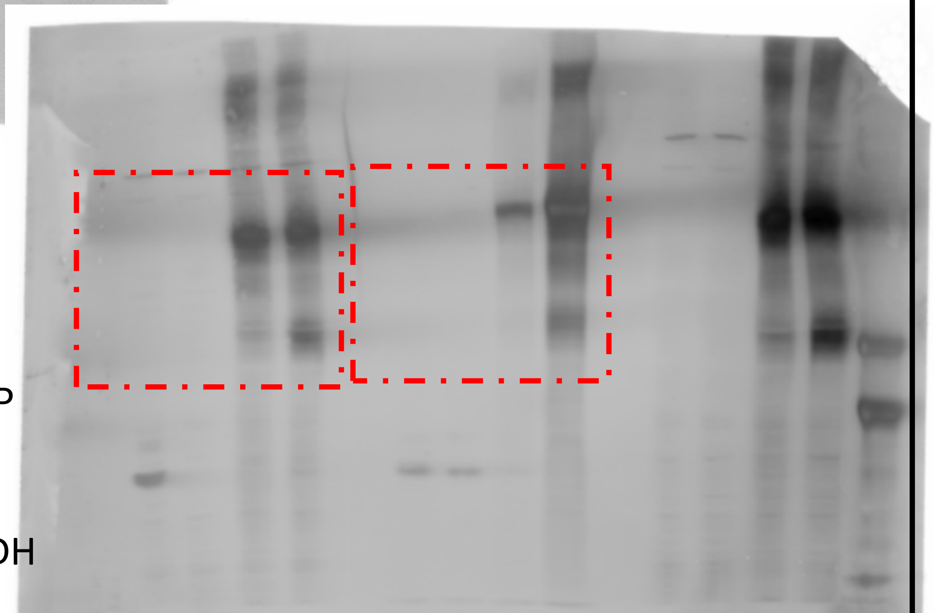

WB: FLAG

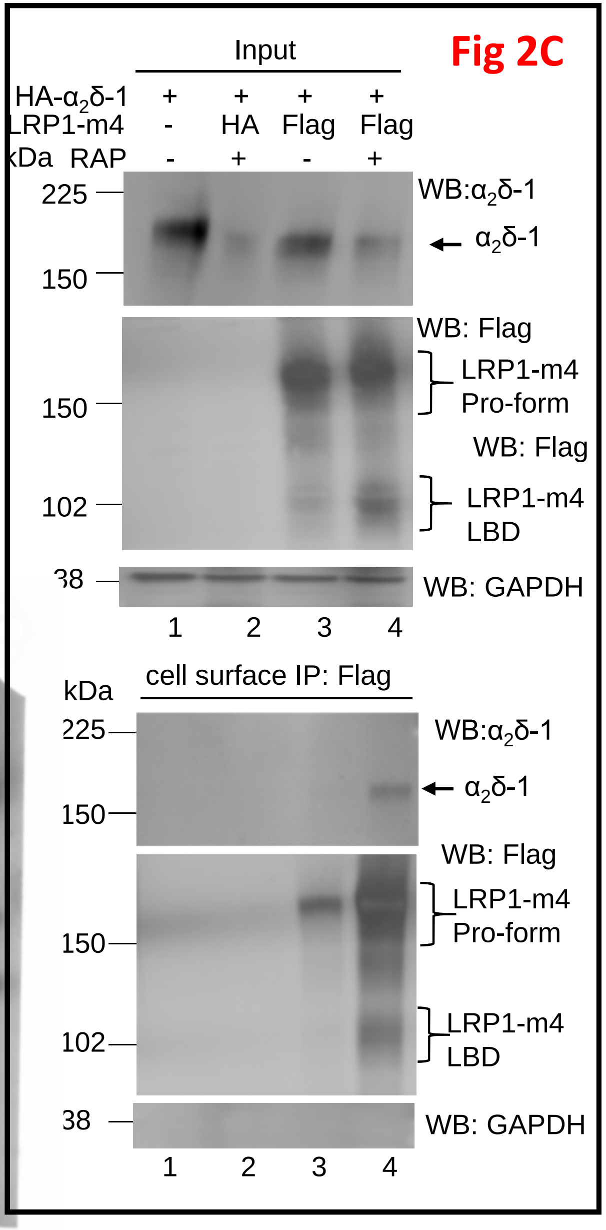

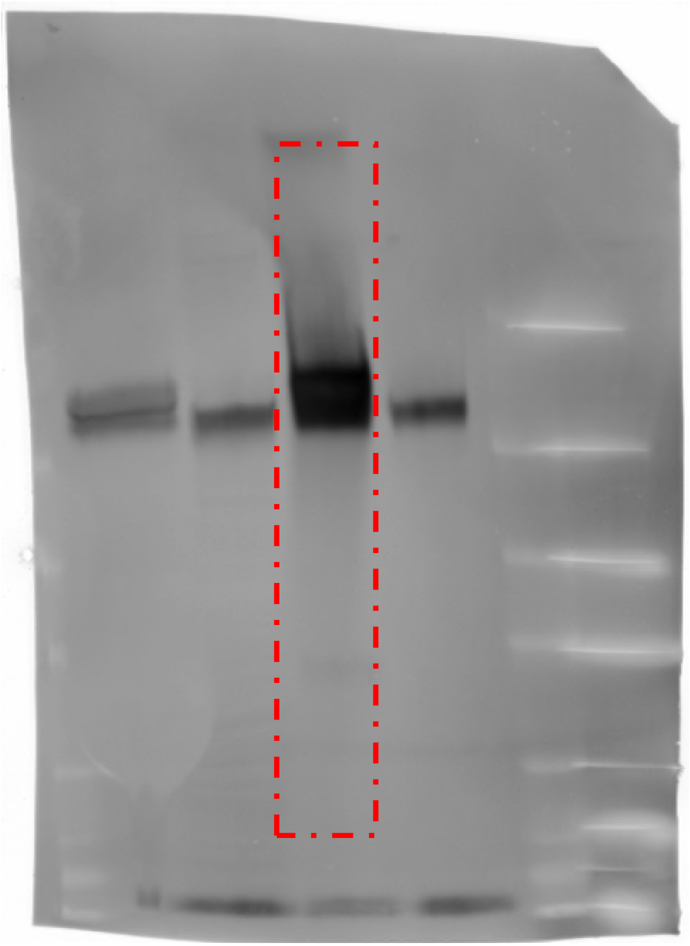

WB: HA

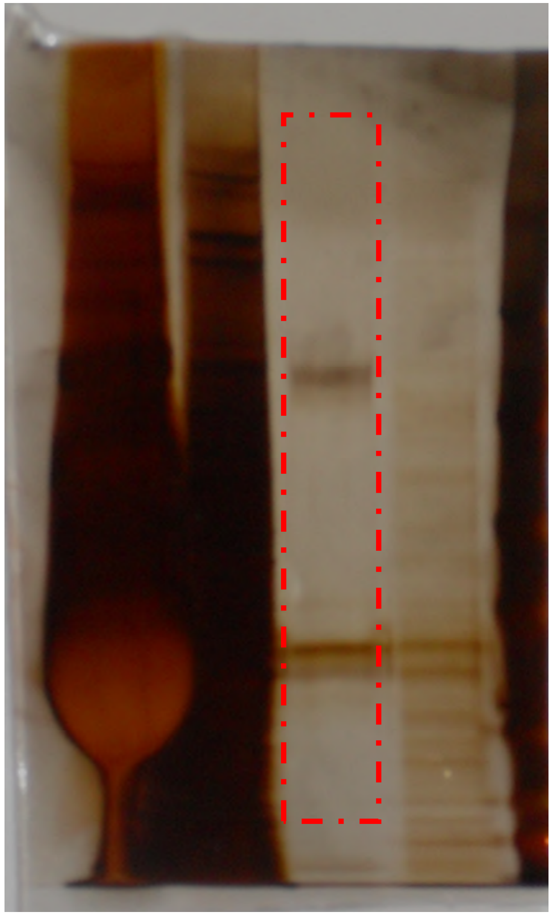

Silver stain

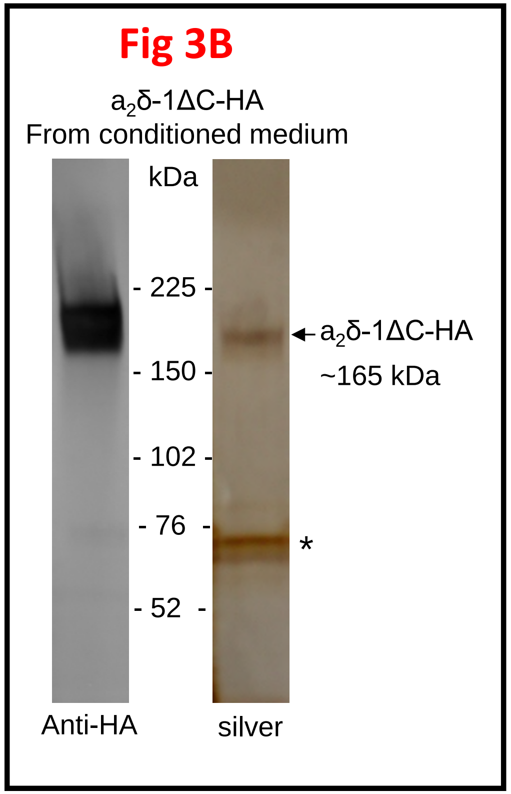

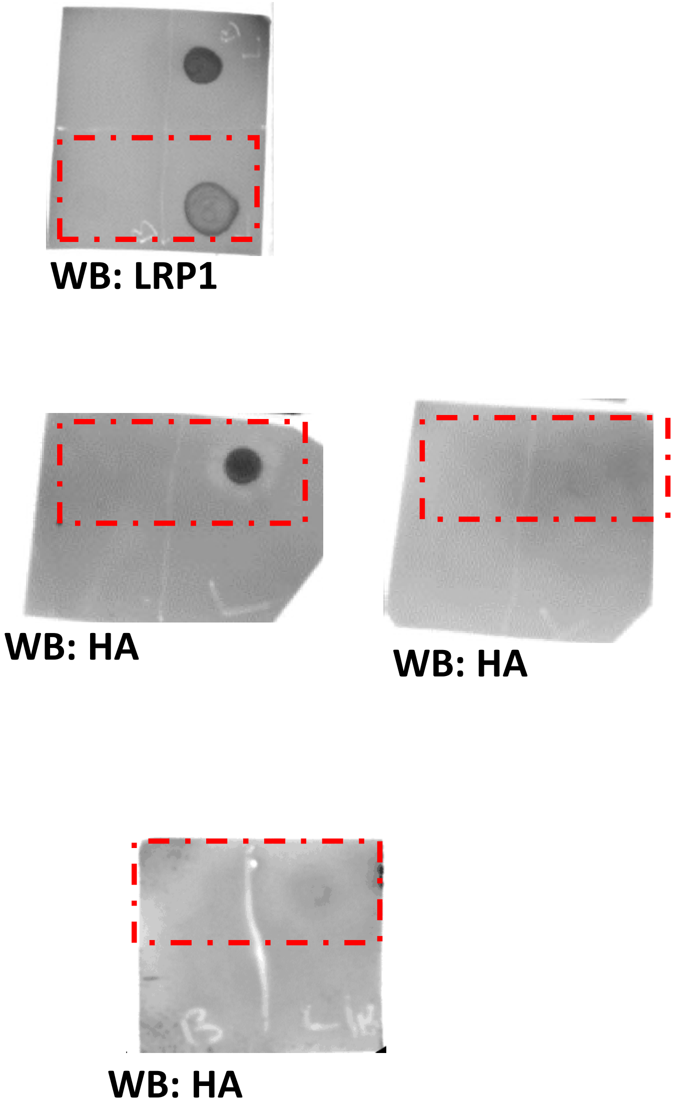
WB: LRP1
WB: HA
WB: HA
WB: HA

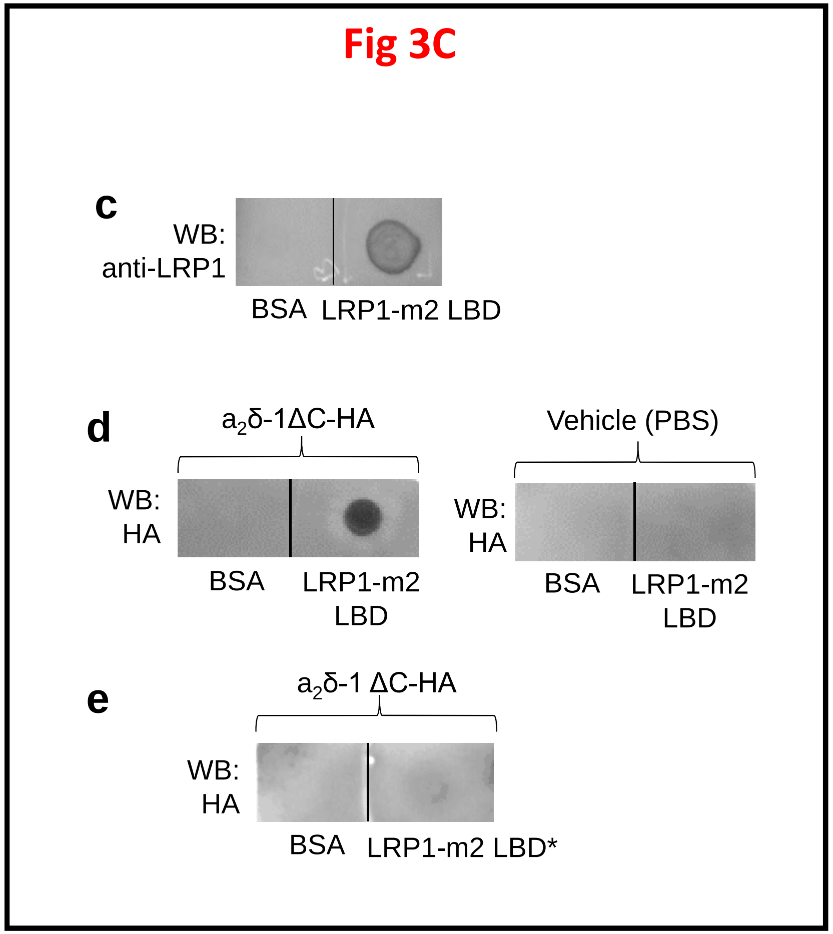
Fig 3C
c
WB:
anti-LRP1
BSA LRP1-m2 LBD
d
a2δ-1ΔC-HA
Vehicle (PBS)
WB:
HA
WB:
HA
BSA LRP1-m2 LBD
BSA LRP1-m2 LBD
e
a2δ-1 ΔC-HA
WB:
HA
BSA LRP1-m2 LBD*

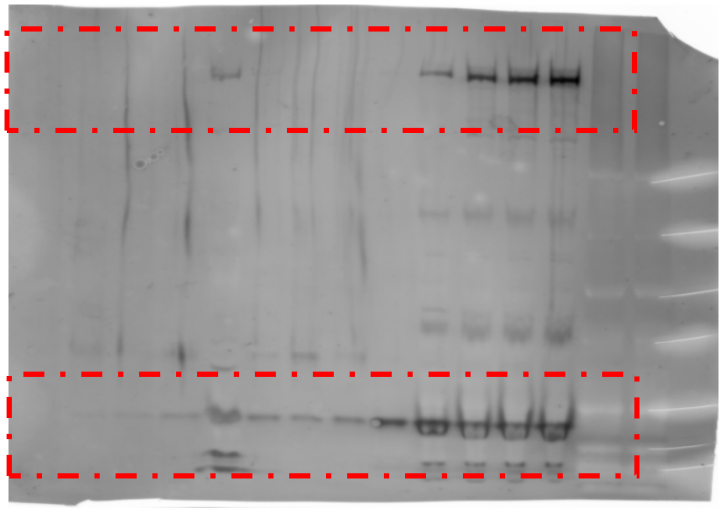

WB: LRP1 (above) and RAP (below)
same Ab stains both (see Mat & Methods)

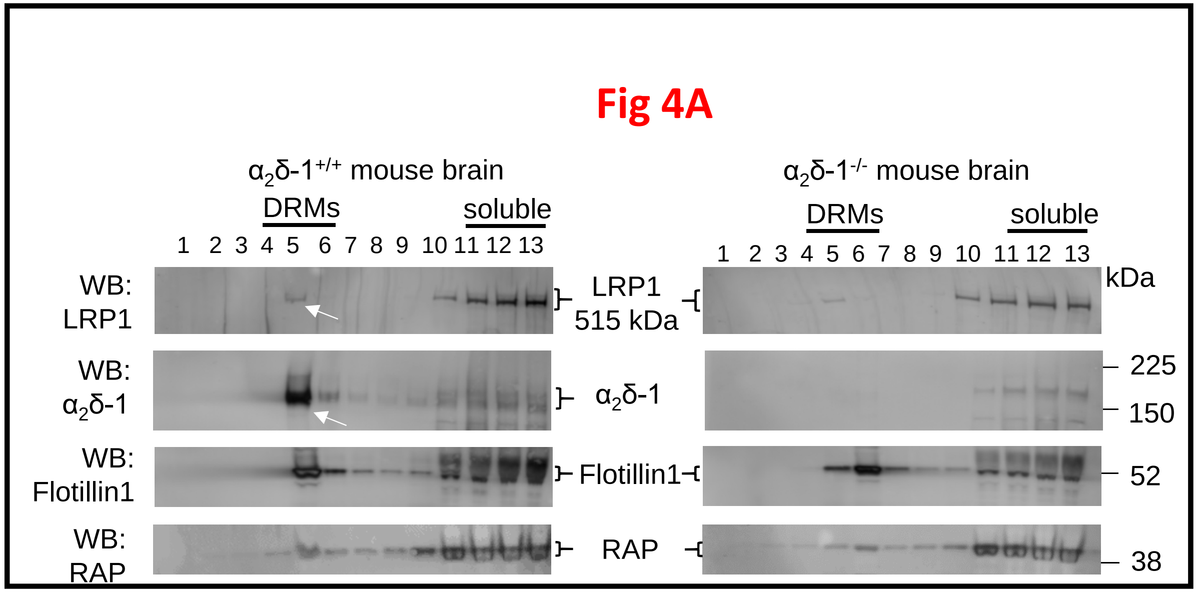

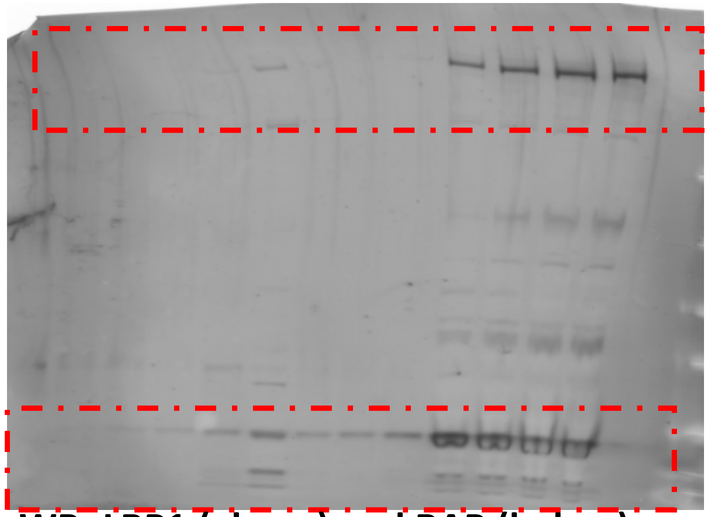

WB: LRP1 (above) and RAP (below)
same Ab stains both (see Mat & Methods)

WB: a2-1

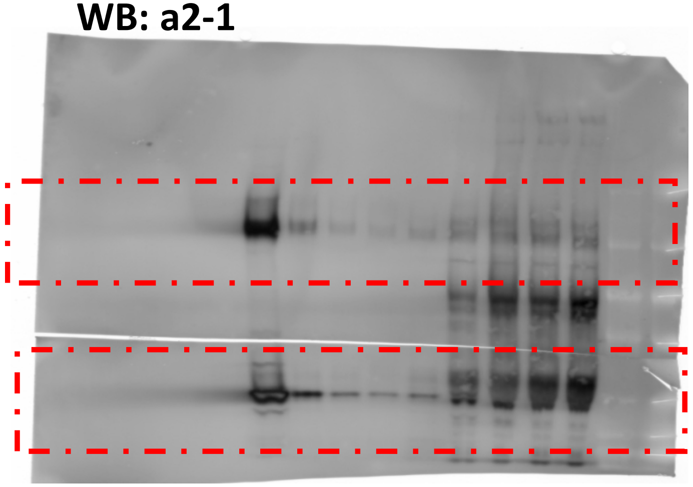

WB: flotillin 1

WB: a2-1

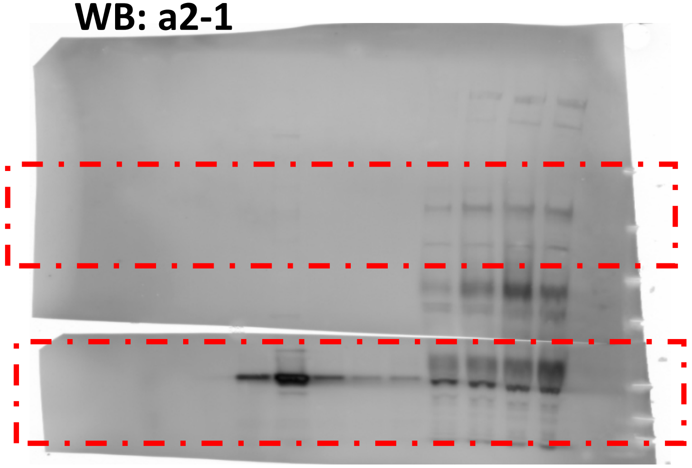

WB: flotillin 1

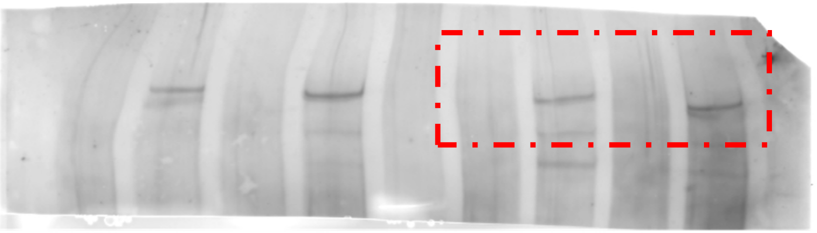

WB: LRP1

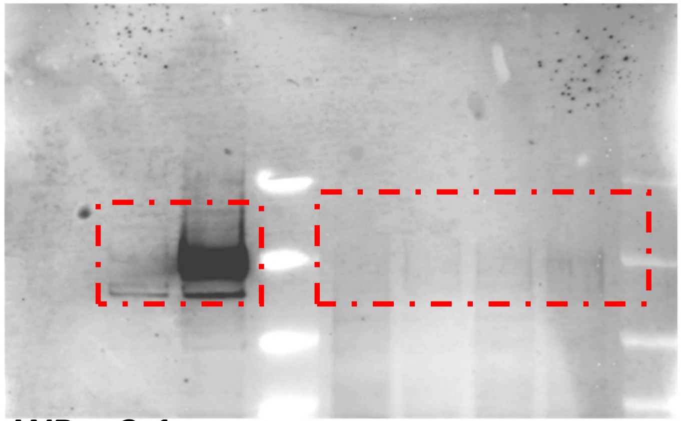

WB: a2-1

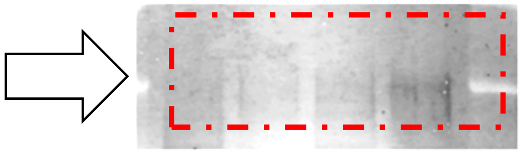

WB: a2-1 (increased contrast)

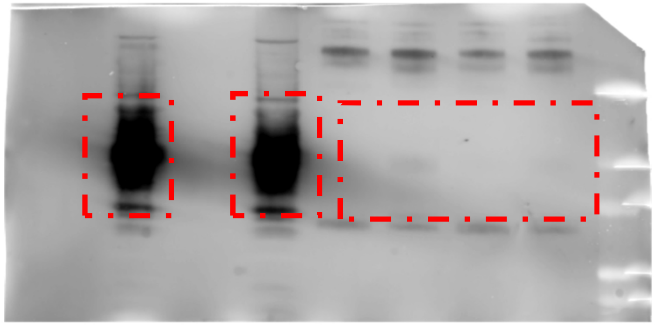

WB: Prp

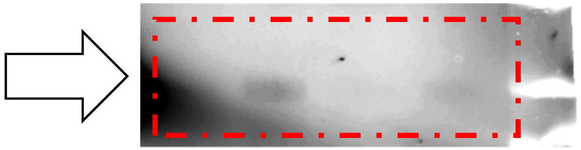

WB: Prp (increased contrast)

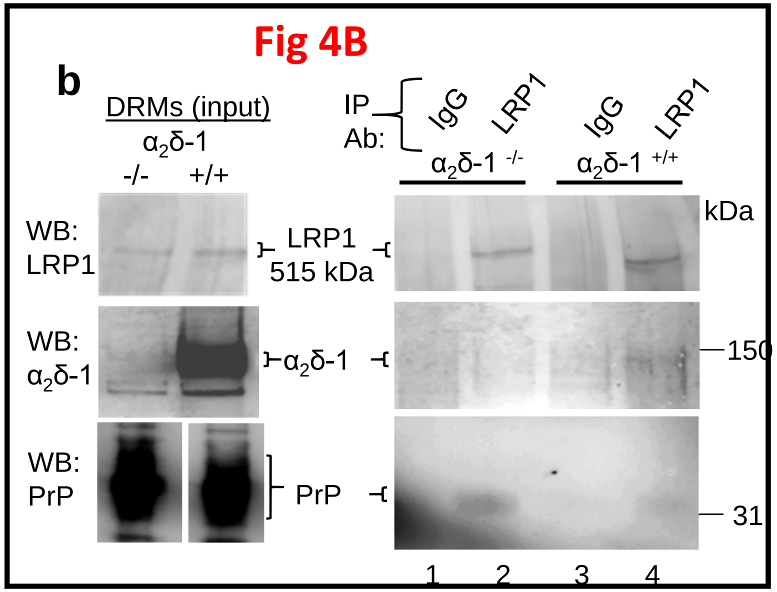

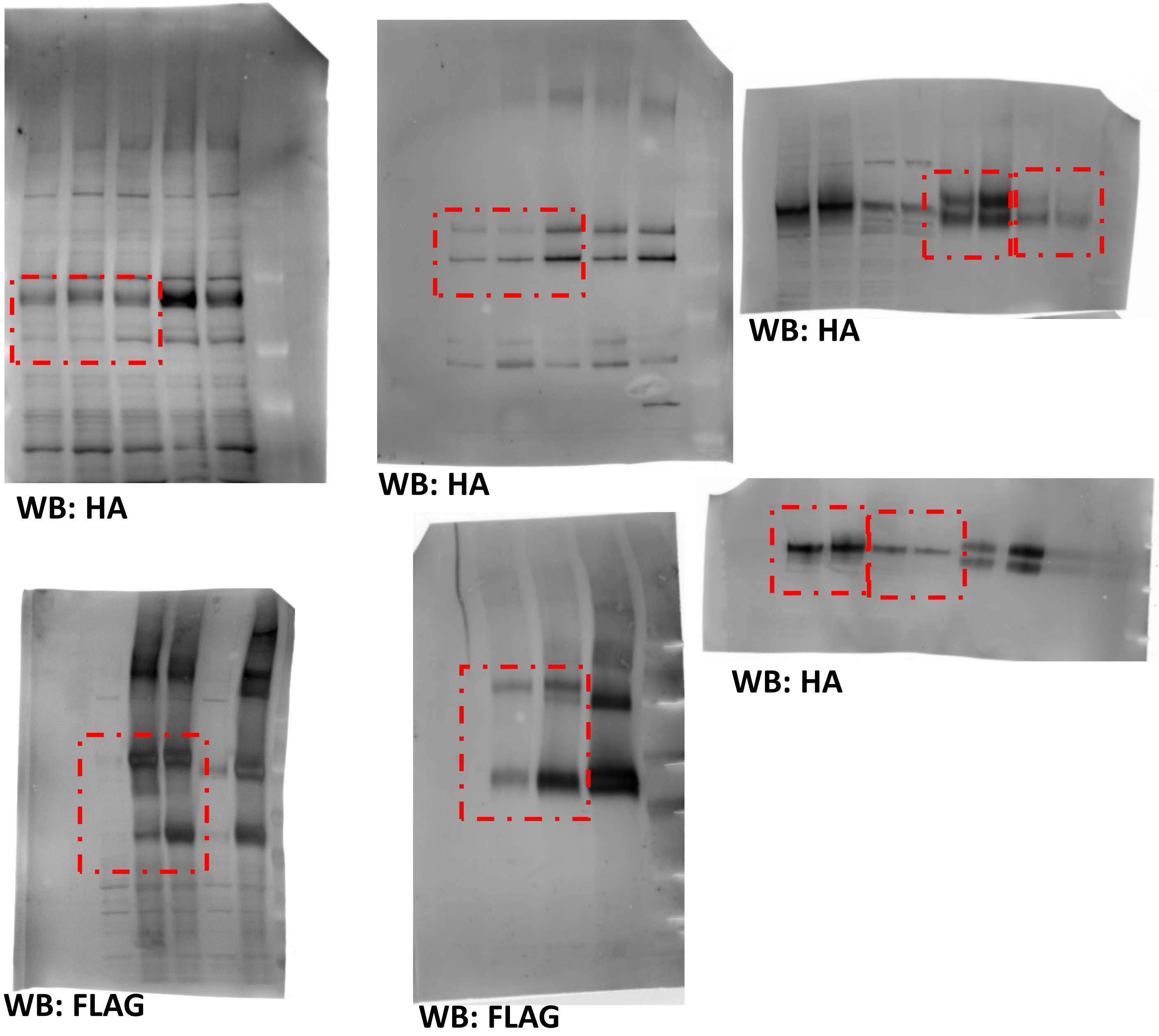
WB: HA
WB: HA
WB: HA
WB: FLAG
WB: FLAG
WB: HA

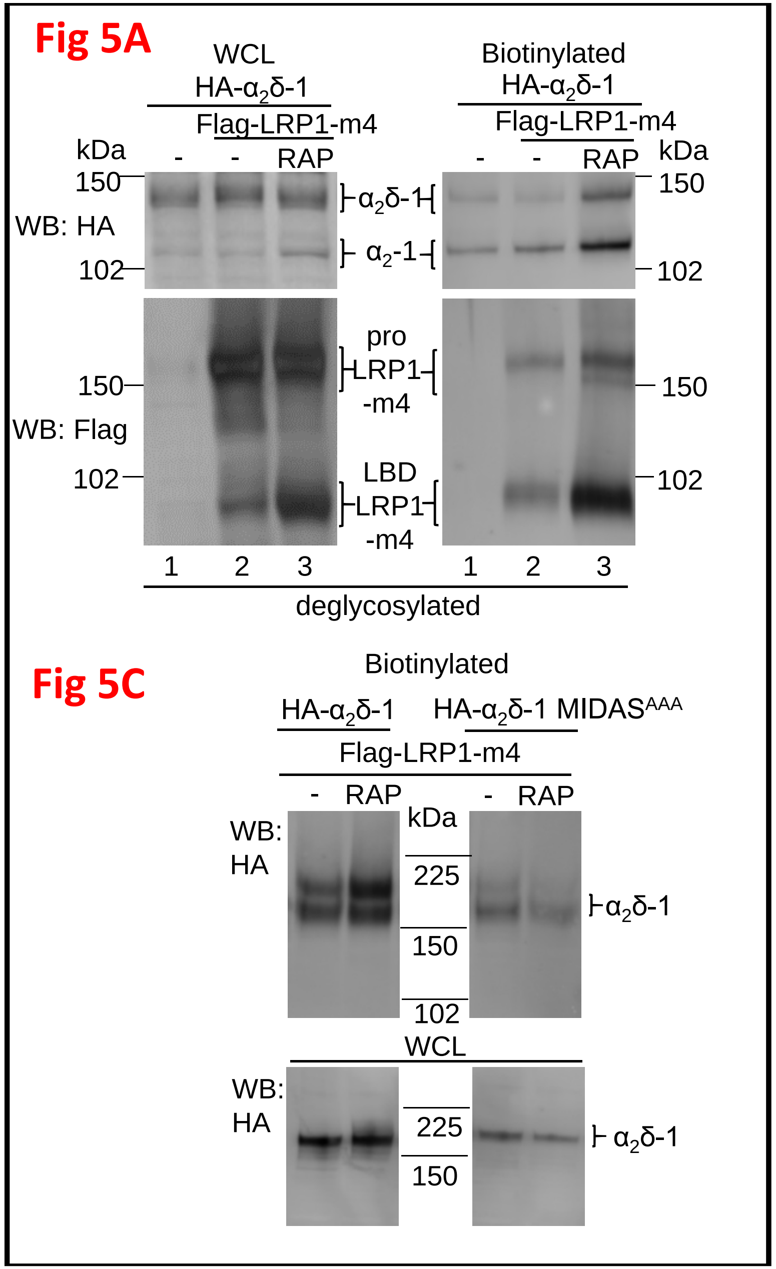
Fig 5A
WCL
HA-α2δ-1
Flag-LRP1-m4
kDa
- - RAP
150
102
WB: HA
α2δ-1
α2-1
Biotinylated
HA-α2δ-1
Flag-LRP1-m4
- - RAP
kDa
150
102
pro
LRP1
-m4
LBD
LRP1
-m4
WB: Flag
150
102
1 2 3
1 2 3
deglycosylated
Fig 5C
Biotinylated
HA-α2δ-1
HA-α2δ-1 MIDASAAA
Flag-LRP1-m4
- RAP
- RAP
WB: HA
kDa
225
150
102
α2δ-1
WCL
WB: HA
225
150
α2δ-1

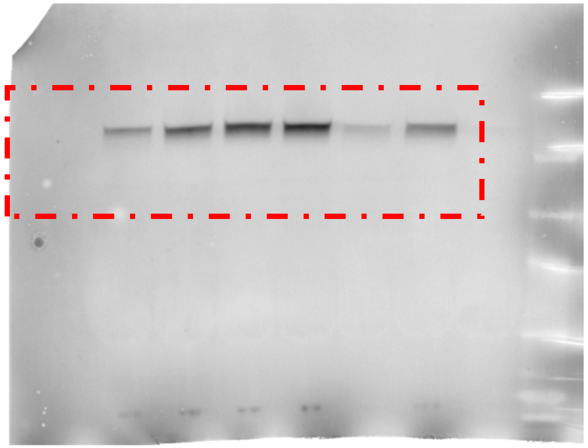

WB: HA

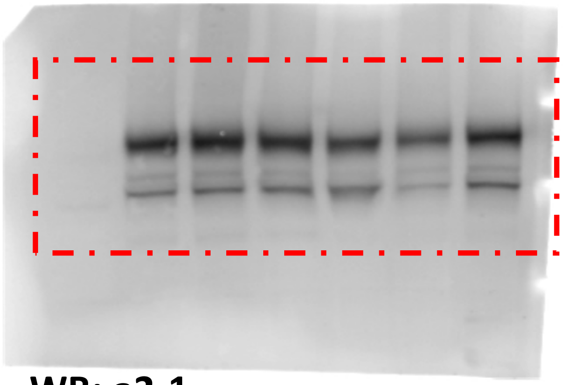

WB: a2-1

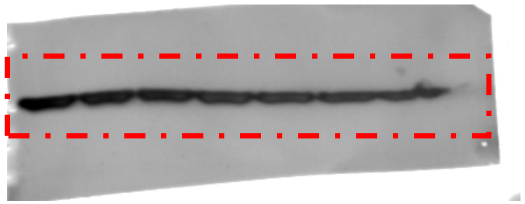

WB:GAPDH

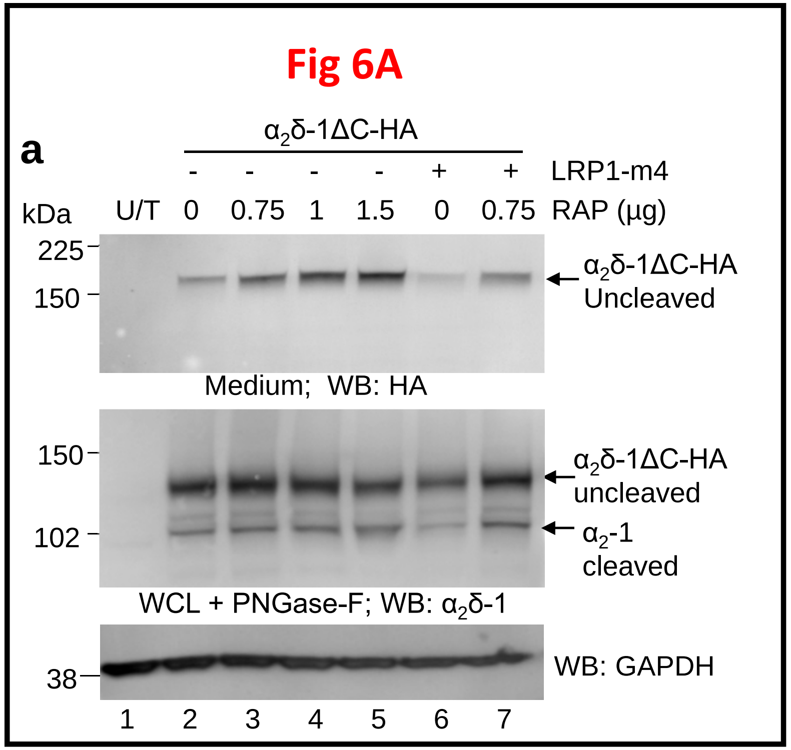

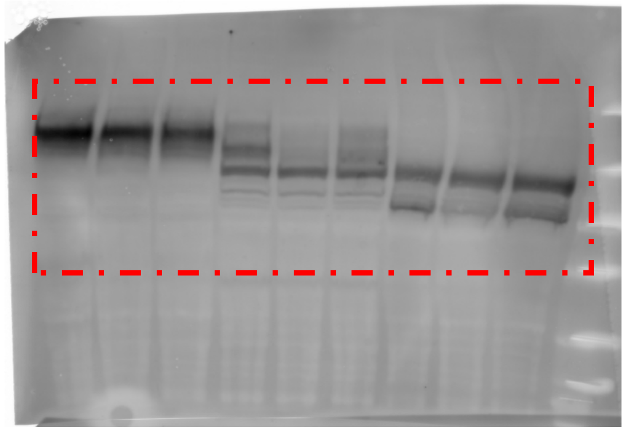

WB: a2-1

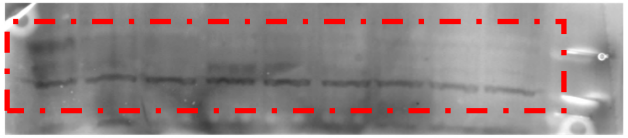

Reblot: Akt

Fig 7B

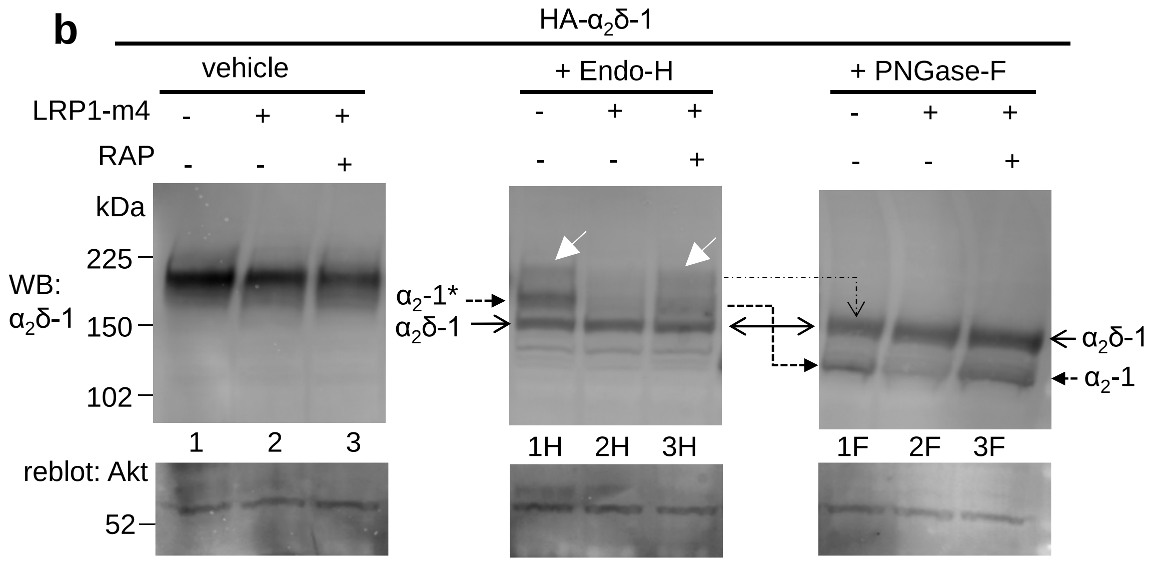

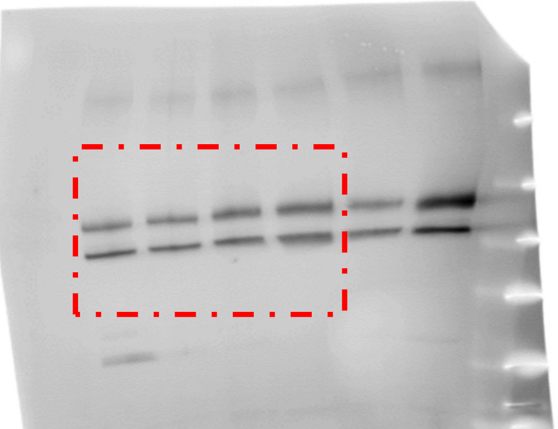

WB: HA

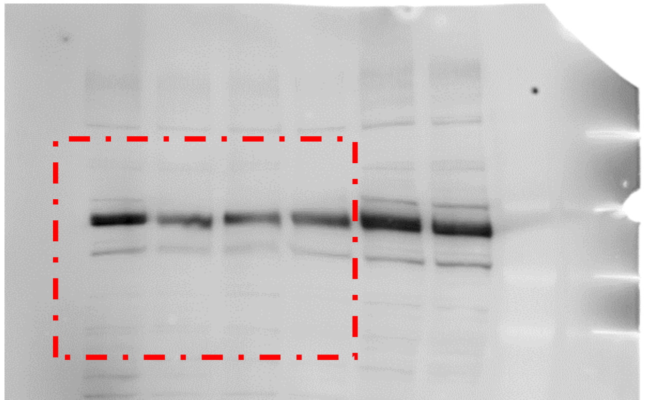

WB: HA

Fig 9E

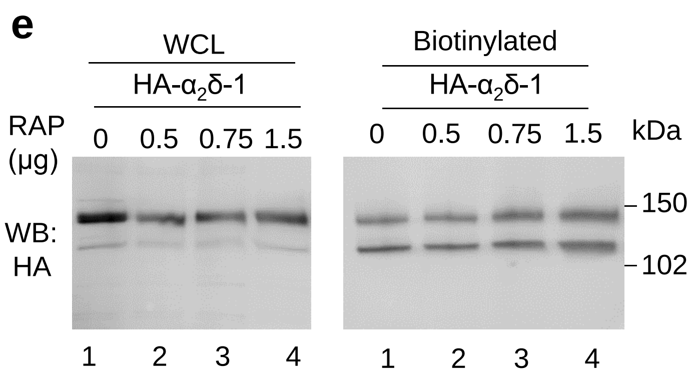

Supplement: Supplementary Information [file srep43802-s1.pdf]
